# Structure–function relationships of family GH70 glucansucrase and 4,6-α-glucanotransferase enzymes, and their evolutionary relationships with family GH13 enzymes

**DOI:** 10.1007/s00018-016-2245-7

**Published:** 2016-05-07

**Authors:** Xiangfeng Meng, Joana Gangoiti, Yuxiang Bai, Tjaard Pijning, Sander S. Van Leeuwen, Lubbert Dijkhuizen

**Affiliations:** 1 Microbial Physiology, Groningen Biomolecular Sciences and Biotechnology Institute (GBB), University of Groningen, Nijenborgh 7, 9747 AG Groningen, The Netherlands; 2 Biophysical Chemistry, Groningen Biomolecular Sciences and Biotechnology Institute (GBB), University of Groningen, Nijenborgh 7, 9747 AG Groningen, The Netherlands

**Keywords:** GH70, GH13, Glucansucrase, 4,6-α-Glucanotransferase, Structure–function, Evolution

## Abstract

Lactic acid bacteria (LAB) are known to produce large amounts of α-glucan exopolysaccharides. Family GH70 glucansucrase (GS) enzymes catalyze the synthesis of these α-glucans from sucrose. The elucidation of the crystal structures of representative GS enzymes has advanced our understanding of their reaction mechanism, especially structural features determining their linkage specificity. In addition, with the increase of genome sequencing, more and more GS enzymes are identified and characterized. Together, such knowledge may promote the synthesis of α-glucans with desired structures and properties from sucrose. In the meantime, two new GH70 subfamilies (GTFB- and GTFC-like) have been identified as 4,6-α-glucanotransferases (4,6-α-GTs) that represent novel evolutionary intermediates between the family GH13 and “classical GH70 enzymes”. These enzymes are not active on sucrose; instead, they use (α1 → 4) glucans (i.e. malto-oligosaccharides and starch) as substrates to synthesize novel α-glucans by introducing linear chains of (α1 → 6) linkages. All these GH70 enzymes are very interesting biocatalysts and hold strong potential for applications in the food, medicine and cosmetic industries. In this review, we summarize the microbiological distribution and the structure–function relationships of family GH70 enzymes, introduce the two newly identified GH70 subfamilies, and discuss evolutionary relationships between family GH70 and GH13 enzymes.

**Electronic supplementary material:**

The online version of this article (doi:10.1007/s00018-016-2245-7) contains supplementary material, which is available to authorized users.

## Introduction

Lactic acid bacteria (LAB) are known to produce lactic acid from sugar metabolism and have been used for the production of traditional dairy products since ancient times. Nowadays, LAB are exploited as starter cultures to improve the preservation, nutritional values, taste and mouthfeel of fermented foods [1]. Various strains are also proven probiotics due to their beneficial effects on human health [2]. LAB have the “generally regarded as safe” (GRAS) status, and are known to produce extracellular polysaccharides (EPS) [3–6]. These EPS are likely involved in the protection of LAB from harsh environmental conditions such as desiccation, osmotic stress and the presence of antimicrobial factors [6–8]. Some EPS facilitate the adhesion of bacterial cells to surfaces and, thus, help LAB to colonize different environments through biofilm formation [6–8]. The EPS produced by *Streptococcus mutans* are important pathogenic factors in dental caries [9, 10]. These EPS have also found valuable applications in food and medicine, and in the cosmetic industries [3–8]. As food supplements, EPS of LAB are explored as texturizers, emulsifiers and viscosifiers and they also hold potential health beneficial effects as dietary fiber and prebiotics [5, 7, 8]. Depending on their composition, EPS are divided into two groups: heteropolysaccharides and homopolysaccharides [11]. Heteropolysaccharides of LAB contain different types of monosaccharides (e.g., glucose, galactose and rhamnose), while homopolysaccharides of LAB consist of only one type of monosaccharide (glucose or fructose). The biosynthesis of heteropolysaccharides is complex and requires the combined action of a large number of proteins including enzymes, transporters and regulators [11, 12]. Generally, Leloir glycosyltransferase enzymes are involved that require expensive nucleotide-activated sugars (e.g., UDP-glucose) [11, 12]. On the contrary, homopolysaccharides are generally synthesized from sucrose using a single glucansucrase (GS) or fructansucrase enzyme [13].

The GS enzymes of LAB belong to glycoside hydrolase family 70 and catalyze the synthesis of α-glucan homopolysaccharides from sucrose [11, 14–16]. Recently, two new GH70 subfamilies have been established, including the GTFB-like 4,6-α-glucanotransferases (GTFB-like 4,6-α-GTs) and GTFC-like 4,6-α-glucanotransferases (GTFC-like 4,6-α-GTs) [17, 18]. They both synthesize novel α-glucans, but use (α1 → 4) linked glucans (i.e. malto-oligosaccharides and amylose) as substrates, and are inactive with sucrose. α-Glucan products synthesized by family GH70 enzymes (GSs, GTFB-like 4,6-α-GTs and GTFC-like 4,6-α-GTs) differ in size, type of linkages, and degree of branching, hence in their physico-chemical properties [13, 16]. This review surveys the recent developments in family GH70 enzyme research with emphasis on their microbiological distribution, the structure–function relationships of GSs and the 2 newly identified GH70 subfamilies, and the evolutionary relationships between family GH70 and GH13 enzymes.

## Microbiological distribution of GH70 GS enzymes

Sucrose, also known as table sugar, is one of the most abundant carbohydrates consumed in our daily life. It is a disaccharide with the formula C_12_H_22_O_11_, consisting of the monosaccharides glucose (d-glucopyranose) and fructose (d-fructofuranose), linked by an (α1↔β2) glycosidic linkage. Early in 1861, Pasteur found a microorganism-derived substance being responsible for the gelification of sugarcane syrups and it was named “dextran” [19, 20]. Van Tiehem isolated this microorganism and named it as *Leuconostoc mesenteroides* in 1878 [19, 20]. An enzyme from the cell-free supernatant was found to be responsible for the synthesis of dextran [21, 22]. Now, dextran is defined as a homopolysaccharide which is composed of d-glucose residues with (α1 → 6) linkages in the main chain and different degrees of (α1 → 2) or (α1 → 3) branched glucosyl units. The GS enzyme that synthesizes dextran is named accordingly as dextransucrase (EC 2.4.1.5).

GSs are exclusively found in LAB, such as *Leuconostoc*, *Streptococcus*, *Lactobacillus* and *Weissella* [15]. GSs are extracellular enzymes and depending on the particular bacterial source, they are produced either as cell wall-attached or free enzymes in culture fluids, or both [23–25]. A diversity of GSs has been characterized from various LAB and were found to produce α-glucans with all the possible glycosidic linkages [(α1 → 2), (α1 → 3), (α1 → 4) and (α1 → 6)], each enzyme with its own linkage specificity. With the fast development of genome sequencing, the number of GSs annotated is rapidly increasing. By Nov. 2015, 264 GSs had been annotated, including 57 characterized in the family GH70 of Carbohydrate-Active Enzymes Database (CAZy; see http://www.cazy.org). GSs are mainly found within the genera *Leuconostoc* (64 of 264), *Streptococcus* (154 of 264), and *Lactobacillus* (23 of 264). Some GSs are also present in other LAB, i.e. *Weissella* (22 of 264) and *Oenococcus* (1 of 264). Some LAB strains produce more than one GS enzyme. For example, *Streptococcus mutans*, which is the main pathogen responsible for dental caries, produces three distinct GSs (GTFB, GTFC and GTFD) (Table 1) [26, 27]. Six different GSs (DSRA, DSRB, DSRE, DSR-DP, DSR-M and BRS-A) are found in the genome of *Leuconostoc citreum* NRRL B-1299 NRRL B-1299 (originally *Leuconostoc mesenteroides* NRRL B-1299) (Table 1) [28]. Several other strains, i.e. *Leuconostoc mesenteroides* NRRL B-1355 (Table 1) [29] and *Streptococcus sobrinus* were also found to contain multiple GSs [30]. These multiple GS enzymes generally display different product (linkage) specificity (Table 1), but it has remained unclear whether they have different physiological roles. Detailed transcriptomic analysis may reveal their individual in vivo roles. Recently, many α-glucan-producing LAB strains have been isolated from fermented food or sugar syrups and were found to possess GSs. In a recent study, a total of thirty LAB from French traditional sourdoughs have been screened for the diversity of exopolysaccharides produced from sucrose [23]. These LAB are mainly *Leuconostoc* and *Weissella* strains. They were found to produce glucans with various glycosidic linkages [(α1 → 2), (α1 → 3) and (α1 → 6)] and the presence of GS-encoding genes was confirmed [23].

**Table 1:** Examples of GS enzymes characterized from different LAB and the glycosidic linkage composition of their α-glucan products

Species	Strains	Enzymes	Glucans	Linkage composition (%)				References
				(α1 → 6)	(α1 → 3)	(α1 → 4)	(α1 → 2)	
*Leuconostoc*	*mesenteroides* NRRL B-512F	DSRS	Dextran	95	5			[138]
	*citreum* NRRL B-1299^a^	DSRA	Dextran	85	15			[38]
		DSRB	Dextran	95	5			[39]
		DSRE	Dextran	81	10	3	5	[37]
		DSR-DP	Dextran	100				[28]
		DSR-M	Dextran	100				[28]
		BRS-A^b^	(α1 → 2)				34	[28]
	*mesenteroides* NRRL B-1355	ASR	Alternan	57	43			[40]
	*citreum* B/110-1-2	DSRF	Dextran	93	6	1		[42]
	*mesenteroides* NRRL B-1118	DSRI	Mutan	50	50			[43]
*Streptococcus*	*mutans* GS5	GTFB	Mutan	12	88			[49]
		GTFC	Mutan	15	85			[50]
		GTFD	Dextran	70	30			[26]
	*oralis*	GTFR	Dextran	86	14			[91]
	*downei* Mfe 28	GTF-S	Dextran	90	10			[53]
		GTF-I	Mutan	12	88			[139]
*Lactobacillus*	*reuteri* 121	GTFA	Reuteran	42		58		[58]
	*reuteri* ATCC 55730	GTFO	Reuteran	21		79		[60]
	*reuteri* 180	GTF180	Dextran	69	31			[57]
	*reuteri* MLI	GTFMLI	Mutan	35	65			[57]
	*sakei* Kg15	GTFKg15	Dextran	90	10			[57]
	*fermentum* Kg3	GTFKg3	Dextran	92	8			[57]
	*parabuchneri* 33	GTF33	Dextran	81	19			[57]
*Weissella*	*confusa* 39-2	DSRC39-2	Dextran	97	3			[62]
	*cibaria*	DSRWC	Dextran	100				[63]

^a^ *Leuconostoc mesenteroides NRRL B*-*1299* has been reclassified as *Leuconostoc citreum* NRRL B-1299 [28]

^b^With sucrose as sole substrate, BRS-A does not catalyze polymer synthesis. In the presence of linear dextran (33 mM) as acceptor substrate and 146 mM sucrose as donor substrate; 34 % (α1 → 2) linkages were found in the product mixture [28]

## GS enzymes from the genus *Leuconostoc*

*Leuconostoc* is most often found in fermented food. GS enzymes are widespread in *Leuconostoc* and the expression of GS from *Leuconostoc* is generally induced by sucrose [31]. Using chemical mutagenesis, mutants (*L. mesenteroides* B-512FMC, B-742CA, B-742CB, B-1142C, B-1299C, B-1355CA and B-1355CB), constitutively expressing GSs, were obtained from wild-type strains *L. mesenteroides* NRRL B-512FM, B-742, B-1142, B-1299 and B-1355 [31–33]. These mutant strains produce glucans with identical structures to those of the wild-type organisms. Most of the GSs from *Leuconostoc* strains produce dextran with mainly (α1 → 6) linkages and minor (α1 → 3) branching linkages (Table 1). The dextran produced by DSRS from *L. mesenteroides* NRRL B-512F has been studied most and is widely used in medicine, food and cosmetic industry [34]. Other α-glucans with different structures are also produced by *Leuconostoc* bacteria. For instance, DSRE from *L. citreum* NRRL B-1299 is a unique enzyme that synthesizes dextran with (α1 → 2) branching linkages (Table 1) [35]. The molecular characterization of this enzyme showed the presence of two catalytic domains (CD1 and CD2), separated by a central glucan-binding domain [35]. Biochemical studies showed that CD1 catalyzed the synthesis of the glucan main chain with predominantly (α1 → 6) linkages, whereas CD2 formed (α1 → 2) branch points on the (α1 → 6) main chain [35–37]. *L. citreum* NRRL B-1299 also produces two additional GSs (DSRA, DSRB). DSRA synthesizes an α-glucan with both (α1 → 6) and (α1 → 3) linkages (Table 1) [38], while the α-glucan produced by DSRB contains larger amounts of (α1 → 6) linkages (Table 1) [39]. The genome sequence analysis of *L. citreum* NRRL B-1299 strain revealed the presence of three more GSs (DSR-DP, BRS-A and DSR-M) [28]. DSR-DP and DSR-M mainly catalyze the synthesis of α-glucans with (α1 → 6) linkages, while BRS-A introduces (α1 → 2) branching linkages [28]. Interestingly, *L. mesenteroides* NRRL B-1355 produces a GS (ASR) (Table 1) [29, 40], synthesizing a glucan with alternating (α1 → 6) and (α1 → 3) linkages [40]. This distinct α-glucan has been named alternan and its enzyme was designated alternansucrase (ASR, EC 2.4.1.140). Recently, more α-glucan-producing *Leuconostoc* bacteria and corresponding GSs have been isolated from various sources. An isolate from sugarcane juice was identified as *Leuconostoc citreum* B/110-1-2 encoding a novel dextransucrase (DSRF) [41]. DSRF synthesizes a dextran with 93 % (α1 → 6) linkages, 6 % (α1 → 3) linkages and 1 % (α1 → 4) linkages (Table 1) [42]. DSRI of *L. mesenteroides* NRRL B-1118 was recently characterized and shown to produce an insoluble α-glucan with approximately 50 % (α1 → 6) and 50 % (α1 → 3) linkages [43]. In a recent report, four and five putative GS genes were identified in the genome sequences of *L. citreum* LBAE-E16 and LBAE-E16, respectively [44].

## GS enzymes from the genus *Streptococcus*

*Streptococcus* strains, especially *S. mutans*, have been recognized as the major dental caries pathogenic bacteria [9, 45]. Dental caries is generally initiated by biofilm formation involving extracellular polysaccharides produced by microorganisms [9, 10]. One of the main components of this biofilm is α-glucan (10-20 % dry weight of biofilm) [46, 47]. The biofilms also trap other bacteria and food debris. Once established, the bacteria in the biofilm ferment sugars and produce acids that cause dental caries [45, 48]. The GSs from *Streptococcus* are constitutively expressed [31]. *S. mutans* produces three distinct GSs (GTFB, GTFC and GTFD) (Table 1) [26, 27, 49, 50]. GTFB (formerly known as GTF-I) and GTFC (GTF-SI) synthesize water-insoluble glucans with large amounts of (α1 → 3) linkages [designated as mutan and its corresponding enzyme as mutansucrase (EC 2.4.1.125)], while GTFD (GTF-S) catalyzes the synthesis of water-soluble glucan with mainly (α1 → 6) linkages [26, 27]. It has been reported that the inactivation of any of the three enzymes resulted in a decrease of smooth-surface carious lesions in the specific-pathogen-free rat model system [51]. In another study, it was demonstrated that GTFB and GTFC play an important role in cellular adherence to smooth surfaces [52]. However, deletion of the *gtf*D gene only slightly affected *S. mutans* in its cellular adherence. Using GTF-deficient *S. mutans* mutants, it was shown that the presence of all 3 GSs at optimum ratio was important for sucrose-dependent adherence [27]. *S. sobrinus* 6715, which is involved in dental caries as well, also contains multiple GSs, producing soluble and insoluble glucans [30]. *Streptococcus downei* Mfe28 also produces two GSs GTF-S and GTF-I (Table 1), being responsible for the synthesis of soluble glucan and non-soluble glucan, respectively [53]. Considering the importance of GSs in the process of dental caries, it has been suggested that specific inhibitors of GS enzymes may be effective for preventing dental caries [9, 27]. However, these specific inhibitors should not inhibit human GH13 enzymes which share high similarity with GH70 enzymes and are essential for the digestion of our carbohydrate food intake.

## GS enzymes from the genus *Lactobacillus*

*Lactobacillus* strains are widely spread in nature and have been used for food application for ages. Some species, e.g., *Lactobacillus reuteri* strains, are considered as probiotic strains due to their beneficial effects for human health [54]. *Lactobacillus* strains were found to produce α-glucans by novel GSs. In an early study, a total of 182 *Lactobacillus* strains were screened for EPS production with sucrose-rich medium; 60 of them were found to produce EPS (glucan or fructan), of which 17 produced large amounts (more than 100 mg/L) [55]. The GSs from *Lactobacillus* are expressed constitutively [13, 25, 56]. Later on, the genes encoding these GSs were cloned and the enzymes were biochemically characterized [56–58]. GTFA from *L. reuteri* 121 synthesizes an α-glucan with 58 % (α1 → 4) linkages and 42 % (α1 → 6) linkages (Table 1) [56]. α-Glucan containing large amounts of (α1 → 4) linkages is referred to as reuteran and its corresponding enzyme as reuteransucrase (EC 2.4.1.-). Structural analysis of reuteran produced by GTFA revealed a large amount of alternating (α1 → 4) and (α1 → 6) linkages [59]. GTFO of the probiotic strain *L. reuteri* ATCC 55730 represents another reuteran-producing GS and its reuteran has an even larger amount of (α1 → 4) linkages (~80 %) compared to GTFA (Table 1) and a smaller amount of (α1 → 6) linkages (~20 %) [60]. A variety of other GSs producing dextran (GTF180, GTFKg15, GTFKg3 and GTF33) and mutan (GTFML1) were also identified in the genus *Lactobacillus* (Table 1) [57]. For example, GTF180 from *L. reuteri* 180 produces an α-glucan with 69 % (α1 → 6) linkages and 31 % (α1 → 3) linkages, while GTFML1 synthesizes an α-glucan with large amounts of (α1 → 3) linkages (~70 %) and (α1 → 6) linkages (~30 %).

## GS enzymes from other lactic acid bacteria

Apart from *Leuconostoc, Streptococcus* and *Lactobacillus*, other LAB (i.e. *Weissella*) also contain genes encoding GS enzymes. *Weissella*, originally included in the genus *Leuconostoc*, also produces EPS from sucrose. Most of the α-glucans produced by *Weissella* strains are dextrans with large amounts of (α1 → 6) linkages (more than 90 %) (Table 1). *Weissella confusa* 39-2 and *Weissella cibaria* LBAE K39 were isolated from wheat sourdoughs and found to produce linear dextrans [61]. The GS (DRSC39-2) from *W. confusa* 39-2 has been characterized [62]. *W. cibaria* isolated from human saliva was found to encode a GS (DSRWC), synthesizing a soluble glucan with large amounts of (α1 → 6) linkages [63]. Also a putative GS gene from *Oenococcus oeni* PSU-1 was identified by genome sequencing [64]. This enzyme and its product remain to be characterized.

## Structures of family GH70 GS enzymes

### Domain organization of family GH70 GS enzymes

**Fig. 1:**
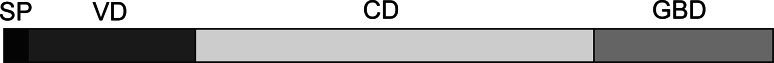
General primary structure of GS proteins from lactic acid bacteria. *SP* signal peptide, *VR* variable region, *CD* catalytic domain, *GBD* glucan binding domain

Based on sequence similarity, GS enzymes are classified as members of glycoside hydrolase family 70 (GH70) in the CAZy database. Prior to the availability of crystal structures of GSs, their catalytic mechanism was mainly explored by comparative studies with GH13 enzymes, which are structurally, evolutionarily and mechanistically related to GH70 family enzymes. GSs are large proteins with an average molecular weight of ~160 kDa. Amino acid sequence analysis showed that all GS proteins have the same domain organization, with only a few exceptions. The amino acid sequences of different GSs contain four different regions [57, 65, 66] (Fig. 1): (a) signal peptide (SP), (b) N-terminal variable region (VR), containing different amino acid repeat units [35, 56, 57, 67, 68], (c) conserved catalytic domain (CD), comprising the catalytic (β/α)_8_ barrel and (d) C-terminal glucan-binding domain (GBD), also consisting of different amino acid repeats and proposed to be involved in α-glucan binding [39, 56, 57, 65, 69, 70].

**Fig. 2:**
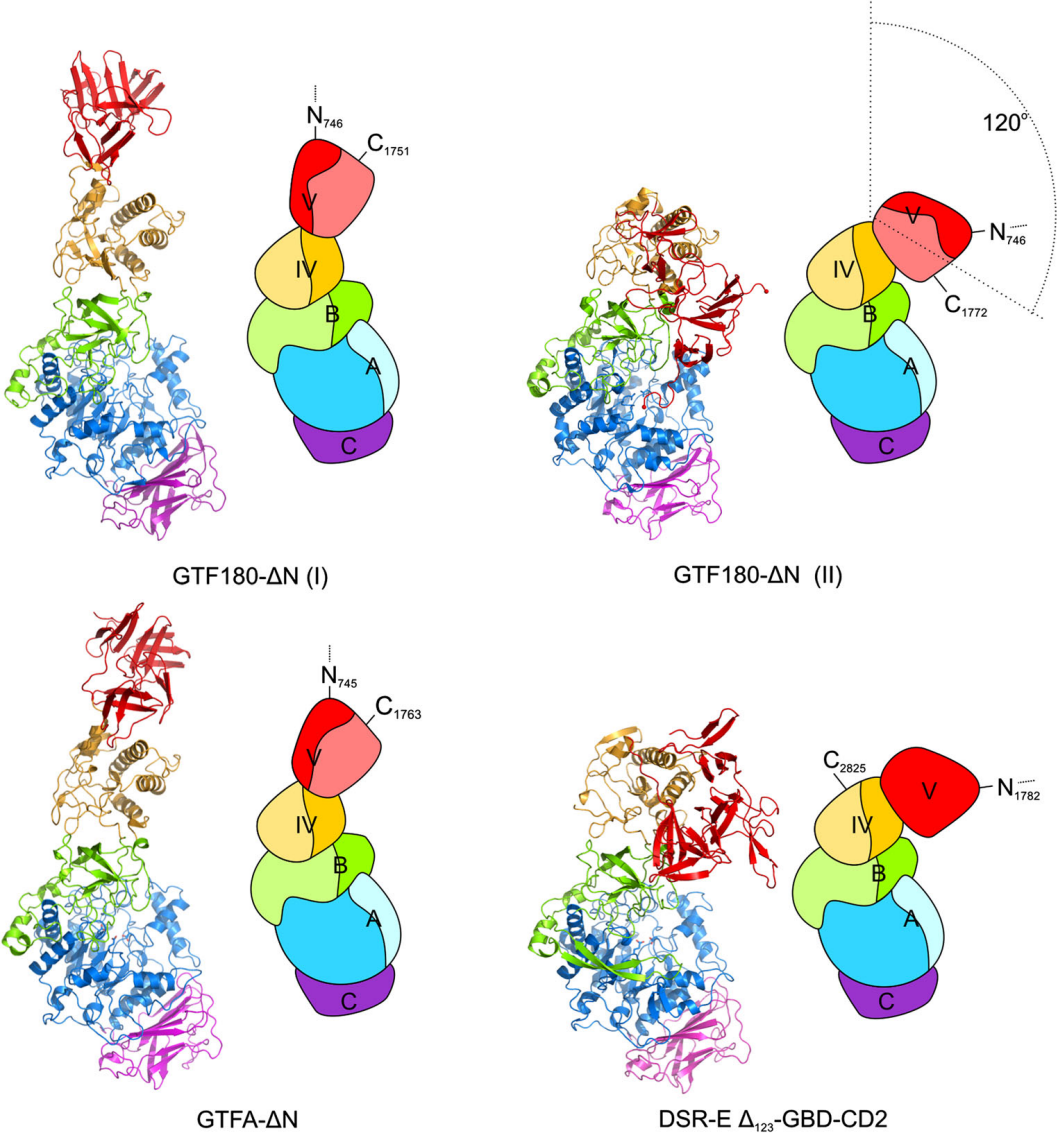
Three-dimensional structures and schematic domain organization of GSs from family GH70. Different domains are *colored* in *blue* (*A*), *green* (*B*), *magenta* (*C*), *yellow* (IV) and *red* (V). Crystal structures [GTF180-ΔN I (PDB: 3KLK, 1.65 Å), GTF180-ΔN II (PDB: 4AYG, 2.0 Å), GTFA-ΔN (PDB: 4AMC, 3.60 Å) and DSR-E ΔN_123_-GBD-CD2 (PDB: 3TTQ, 1.90 Å)] are shown. This figure has been adapted from [15]

Over the years, large efforts have been made to generate crystals of GS proteins suitable for X-ray diffractions studies, but only recently the first 3D structures of GS proteins have been solved [71]. To date, the crystal structures of four GH70 GSs (GTF180-ΔN from *L. reuteri* 180, GTFA-ΔN from *L. reuteri* 121, GTF-SI (amino acid residues 244-1163) from *S. mutans* and ΔN_123_-GBD-CD2 of DSR-E from *L. citreum* NRRL B-1299) have been determined (Fig. 2), revealing a common domain organization [36, 71–73]. In all cases, a truncated enzyme (i.e. lacking the N-terminal variable region, but retaining full activity) was used for crystallization. Rather surprisingly, the 3D structures of these GSs revealed a novel domain organization (Fig. 2), different from the previous prediction based on primary sequence alignments (Fig. 1). The polypeptide chains of the truncated GSs follow a U-shape path to form five domains (A, B, C, IV and V) (Fig. 2) [71]. Except for domain C, each of the four domains is formed by two discontinuous polypeptide chains from both the N- and C-termini. The A, B, and C domains form the catalytic core and are also found in GH13 enzymes (e.g., α-amylase). In contrast, domains IV and V are unique to GH70 GSs.

**Fig. 3:**
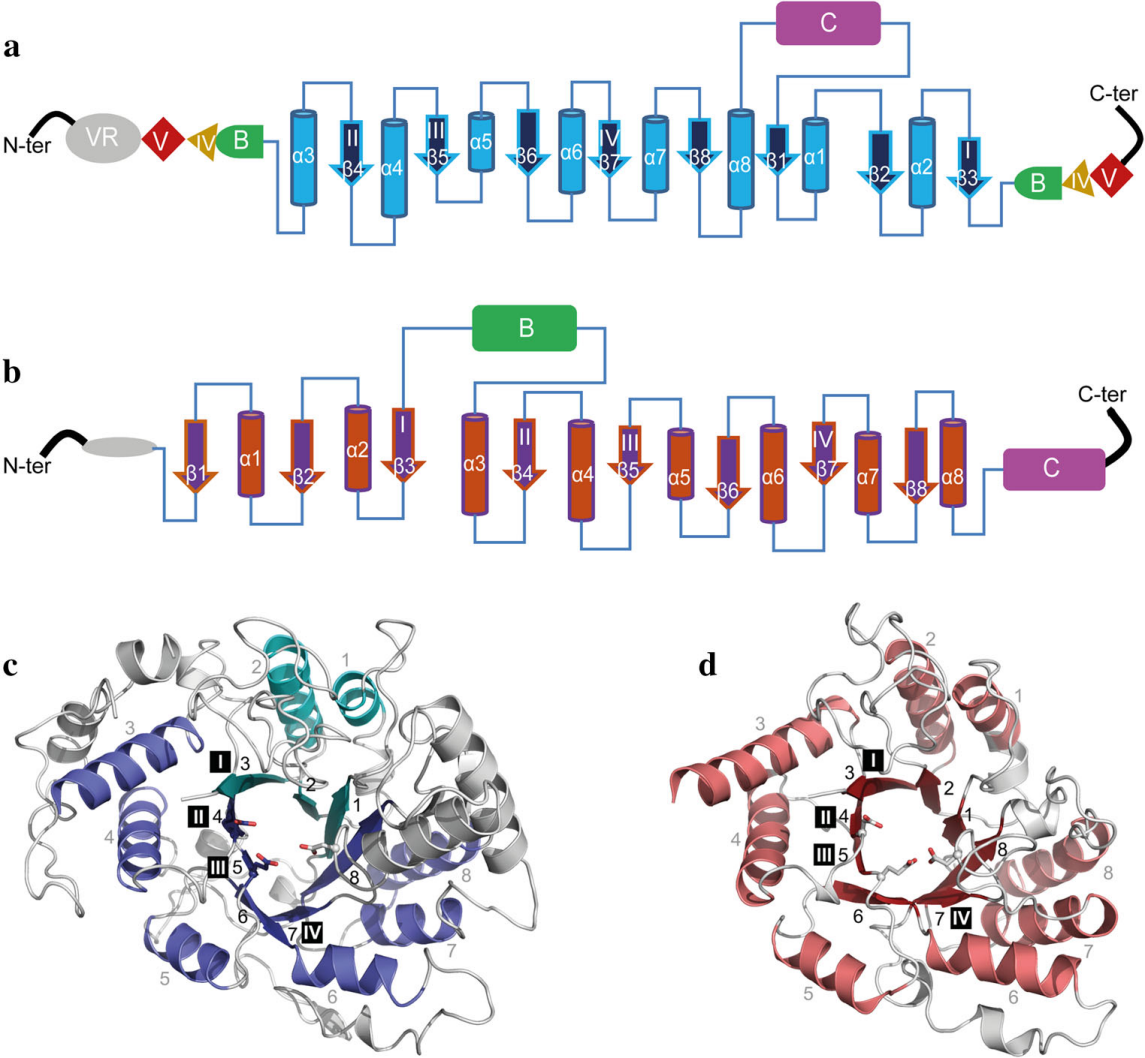
Topology diagrams models of family GH70 GSs with a circularly permutated (β/α)_8_ barrel (**a**) and the family GH13 α-amylase (β/α)_8_ barrel (**b**). Cylinders represent α-helices and arrows represent β-strands. The equivalent α-helices and β-strands in GH70 GSs and GH13 α-amylases are numbered the same. The different domains in GH70 and GH13 enzymes are indicated. Domain C of GH70 GSs is inserted between α-helix 8 and β-strand 1 while that of GH13 family α-amylase locates C-terminally of the (β/α)_8_ barrel. Domain B of GH13 α-amylases is inserted between β-strand 3 and α-helix 3 while that of GH70 GSs is formed by two discontinuous polypeptide segments from both the N- and C-termini. The same is true for domains IV and V of GH70 GSs. A variable region (VR) is present in the N-terminus of GH70 GSs. The four conserved sequence motifs (I–IV) which are located in β-strands 3, 4, 5, and 7, respectively, and are shared between family GH70 GSs and GH13 enzymes, are indicated within the β-strand. The structure of the catalytic domain in the GH70 GSs representative GTF180-ΔN (c, PDB: 3KLK) of *L. reuteri* 180 and in the GH13 representative α-amylase of *Bacillus licheniformis* (d, PDB: 1BPL). The (β/α)_8_ barrel is colored for a better representation. α-Helices and β-strands are numbered, and the conserved sequence motifs (I–IV) are indicated at the corresponding β-strand. The circularly permutated (β/α)_8_ barrel of GH70 GSs is formed by two separate polypeptide segments (N-terminal parts colored in deep blue and C-terminal parts colored in cyan), which is caused by the insertion of domain C

**Fig. 4:**
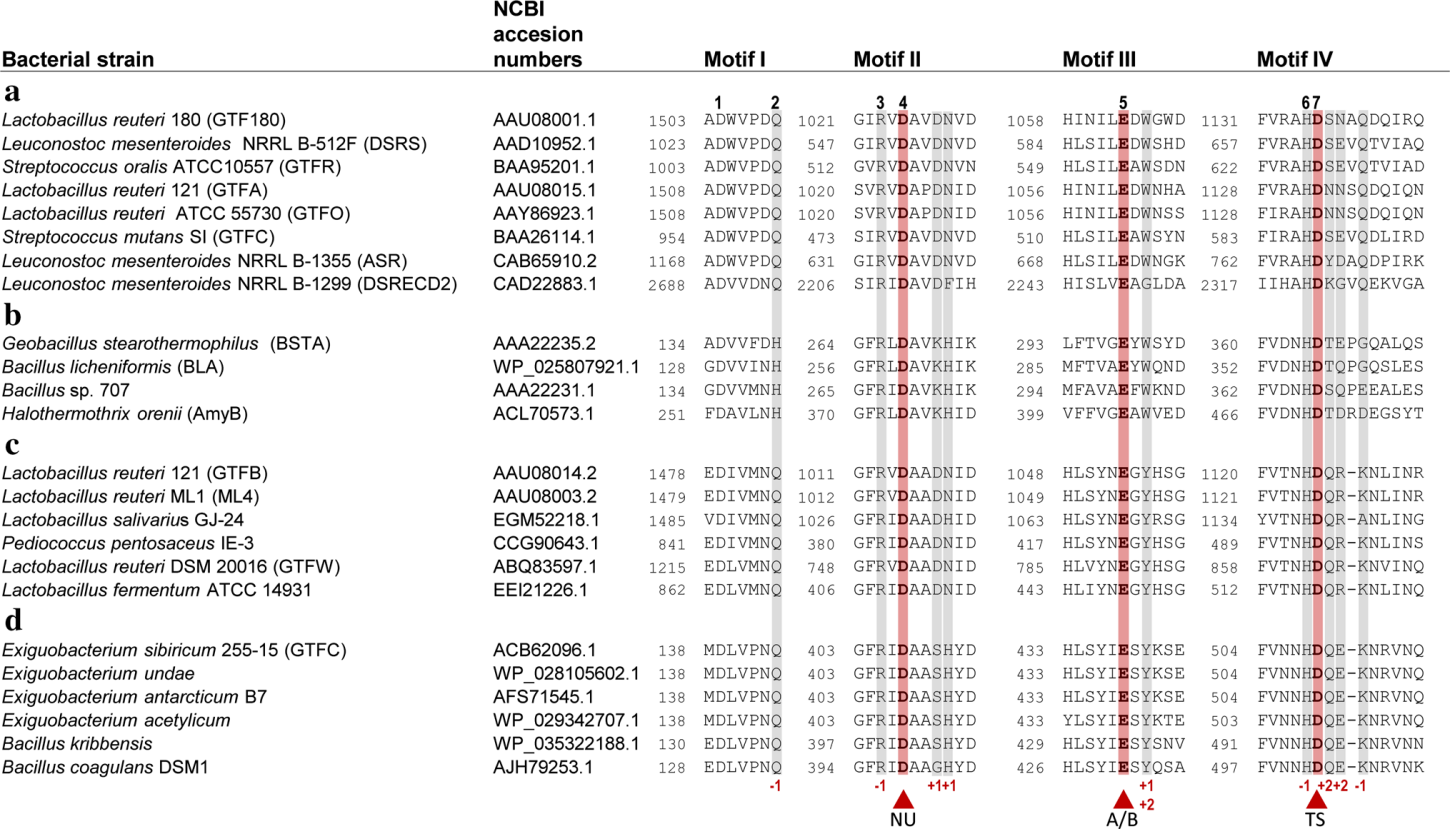
Amino acid sequence alignment of conserved sequence motifs I, II, III, and IV in the catalytic domains of GH70 GSs (**a**), GH13 α-amylases (**b**), (putative) GTFB-like 4,6-α-GTs (**c**) and (putative) GTFC-like 4,6-α-GTs (**d**). The seven strictly conserved amino acid residues in GH13 enzymes (indicated by the *numbers* 1–7 above the sequences) are also highlighted. Six of them are also conserved in the GH70 GSs, novel GH70 GTFB-like 4,6-α-GTs and GTFC-like 4,6-α-GTs. Amino acids that constitute the catalytic triad are highlighted in *red*. Residues forming acceptor binding subsites −1, +1 and +2 in GTF180-ΔN are shown *lightly shaded*. *Symbols* NU = nucleophile, A/B = general acid/base, TS = transition state stabilizer

Domain A contains the circularly permutated (β/α)_8_ barrel in contrast to the (β/α)_8_ barrel in GH13 enzymes (Fig. 3) [74]. The (β/α)_8_ barrel is characterized by the presence of 8 β-strands (β1–β8) residing in the core of the enzyme alternating with 8 α-helices (α1-α8) surrounding the β-strands (Fig. 3). From N-terminus to C-terminus, the circularly permuted (β/α)_8_ barrel of GH70 GSs starts with the α-helix that corresponds with α3 of family GH13 enzymes with the sequence of N terminus-α3-β4-α4-β5-α5-β6-α6-β7-α7-β8-α8-β1-α1-β2-α2-β3-C-terminus (Fig. 3a and 3c) [74]. The four conserved amino acid sequence motifs (I to IV) of the GH13 family enzymes are also present in GH70 GSs (Fig. 4) [56, 75]. Due to the circularly permutated structure of GS enzymes, their conserved motif I is located C-terminal of motifs II to IV (Fig. 3). Six of the seven conserved residues from motifs I to IV in family GH13 are also present in family GH70 (Fig. 4) [13]. Only the His134 (α-amylase of *Bacillus licheniformis* numbering) in family GH13 is replaced by Gln (Gln1509, GTF180 numbering) in family GH70 GSs [76]. Specifically, the three catalytic site residues (the nucleophile Asp1025, acid/base catalyst Glu1063 and transition state stabilizer Asp1136, GTF180 numbering) are located in the loops following β-strands β4, β5 and β7, in the conserved sequence motifs II, III and IV, respectively (Fig. 3) [71]. The mechanism of glycosidic linkage cleavage by GH70 GS enzymes is similar to that of family GH13 α-amylase enzymes (see below).

Two discontinuous inserts with a large stretch of amino acids between β-sheet 3 and α-helix 3 from the N- and C-termini form a separate domain B next to domain A (Figs. 2, 3). The position of domain B in GH70 GS enzymes is similar to that in family GH13 enzymes. The active site of GSs is located in a pocket-shaped cavity and lies at the interface of domain A and domain B [71]. Domain B contains several amino acids (L938, L940, A978 and L981) for shaping the substrate/acceptor binding sites, which may contribute to the enzyme product specificity. [71]. Domain C of GH70 GSs, located at the bottom of the U-shape (Figs. 2, 3), contains an eight-stranded β-sheet with a Greek key motif similar to that of domain C in family GH13 enzymes [71, 77]. It is the only domain in GH70 GS enzymes that is formed by a continuous polypeptide segment. The function of domain C remains unclear, although it is widely distributed within the GH13 and GH70 families.

In addition to domains A, B and C, GH70 GSs have two extra domains (IV and V) attached to the catalytic core. Domain IV lies between domain B and domain V. The structure of domain IV is novel and it has no similarity to any other known protein structure and only occurs in GH70 enzymes [15, 71]. Domain V is located adjacent to domain IV. It contains several sequence repeats which have been shown to be involved in glucan binding [13, 67, 69, 70, 78]. Structural analysis of domain V revealed the presence of a consensus β-solenoid fold with multiple copies [15, 71]. The precise roles of domains IV and V have remained unknown. It has been proposed that domain IV acts as a hinge that facilitates the growth of glucan chain by bringing the glucan chain bound to domain V toward and away from the catalytic site, but no experimental evidence is available yet [72]. Comparison of crystal structures of different GS enzymes revealed a positional variability of domain V (Fig. 2). For example, compared to the crystal structure of GTF180-ΔN, domain V of GTFA-ΔN showed a shift of about 20 Å with respect to the other domains [73]. Surprisingly, domain V of ΔN_123_-GBD-CD2 of DSRE is located in a completely different position adjacent to the catalytic core, adopting a similar fold as domain V of GTF180-ΔN [36]. A B-factor analysis of domain V in the different crystal structures showed that its average value is higher than the other four domains, which indicates that it is more flexible [79]. Recently, the flexibility of domain V was also demonstrated by the elucidation of a new crystal form of GTF180-ΔN with a 120° rotation at a hinge located between domains IV and V (Fig. 2), further supported by the observation of positional flexibility of domain V in solution [79]. Truncation of domain V from GTF180-ΔN did not have significant effects on its linkage specificity [80]. However, higher amounts of oligosaccharides (size ~2 kDa) were produced at the expense of polysaccharide production [80]. This provided direct evidence for the involvement of domain V in polysaccharide synthesis.

Although the crystal structure of a full-length GS is not available yet, small angle X-ray scattering studies have showed that the N-terminal variable region (~700 amino acids) extends further away from domain V [79]. As a result, the overall shape of GTF180 showed an almost symmetric boomerang-like molecular shape with the bend point located between domains IV and V.

## Catalytic mechanism of GS enzymes

**Fig. 5:**
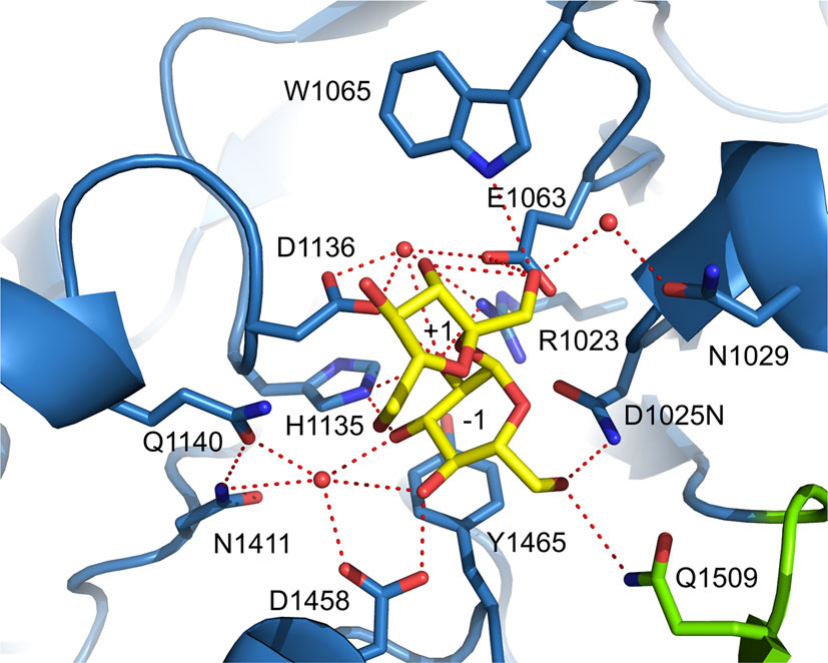
Sucrose (donor substrate) binding site −1 and +1 in the crystal structure of the GTF180-ΔN mutant D1025N sucrose complex (PDB: 3HZ3) [71]. Sucrose is shown with yellow carbon atoms. Residues from domain A (*blue*) and B (*green*) surrounding the −1 and +1 subsites are indicated. Hydrogen bonds are shown as *dashed lines*

Similar to family GH13 enzymes, the α-retaining double displacement reaction mechanism is used by GH70 GSs [13, 65, 77]. This two-step mechanism involves three catalytic residues, the nucleophile and the acid/base catalysts as well as the transition state stabilizing residue (Fig. 4). In the first step, the (α1↔β2) glycosidic linkage of sucrose is cleaved by the attack of the nucleophile with the formation of a β-glucosyl-enzyme covalent intermediate. This glucosyl-enzyme intermediate is stabilized by the transition state stabilizing residue. The acid/base catalyst protonates the fructosyl moiety, resulting in release of fructose. In the subsequent step, the glucosyl moiety is transferred to the non-reducing end of an acceptor with retention of the α-anomeric configuration. Repeating this reaction cycle results in the synthesis of gluco-oligosaccharides and α-glucan polysaccharides from sucrose. The crystal structure of GTF180-ΔN validates that GSs use the same set of amino acids to catalyze the reaction as family GH13 enzymes [71, 77]. The crystal structure of the inactive mutant GTF180-ΔN D1025 N bound with sucrose revealed that the seven strictly conserved residues at the active site, six also employed by GH13 enzymes, make similar interactions with the -1 glucosyl moiety of sucrose (Figs. 4, 5). The glucosyl moiety binds at the -1 site by the conserved interactions with R1023,D1025, H1135, D1136, E1063, Y1465 and Q1509, of which R1023, D1025, H1135, D1136 and Q1509 make direct H-bonds to glucosyl hydroxyl groups. Residues D1025 and E1063 are oriented towards the glycosidic oxygen and the anomeric C1 atom of the glucosyl moiety of sucrose. Residue D1025 acts as the nucleophilic residue which attacks the anomeric C1 carbon of the glucosyl unit of sucrose to form a β-glucosyl-enzyme covalent intermediate, stabilized by the transition state stabilizer (D1136), while residue E1063 is the acid/base catalyst donating a proton to facilitate the release of fructose and deprotonating the acceptor molecule to activate it. Residue Y1465 located at the bottom of subsite −1 provides hydrophobic interactions with the glucosyl moiety of sucrose. The conserved residue D1504 is outside of the catalytic center, making a H-bond to the conserved Y1465 hydroxyl group. Furthermore, subsite −1 is lined by residues Q1140 and N1411, which form hydrogen bonds with the C3 hydroxyl group of the glucosyl moiety through a water molecule. This results in a pocket-shaped active site which can only accommodate one glucosyl moiety, demonstrating that GH70 GSs are able to transfer only one glucosyl unit in each reaction cycle. In contrast, a cleft-shaped active site with contiguous multiple donor subsites is present in family GH13 α-amylases. In GTF180-ΔN, the fructosyl moiety binds more loosely at the +1 subsite, having interactions with residues E1063, W1065, N1029, D1136 and Q1140 (Fig. 5). The C3 hydroxyl group of the fructosyl moiety is involved in a hydrogen bond network with the side chains of W1065, D1136 and E1063, while N1029 and Q1140 make a hydrogen bond with the C1 and C6 hydroxyl groups, respectively. In addition, two hydrophobic residues (L981 and L982) from domain B make Van der Waals’ interactions with the fructosyl moiety [71].

## Mechanism of α-glucan synthesis by family GH70 GS enzymes

Despite the availability of crystal structures, the mechanism of glucan synthesis by GS is not fully understood yet, especially regarding the initiation of α-glucan synthesis and the synthesis process, the mode of action (processive versus non-processive), the formation of branches and the linkage specificity.

### Initiation of α-glucan synthesis

**Fig. 6:**
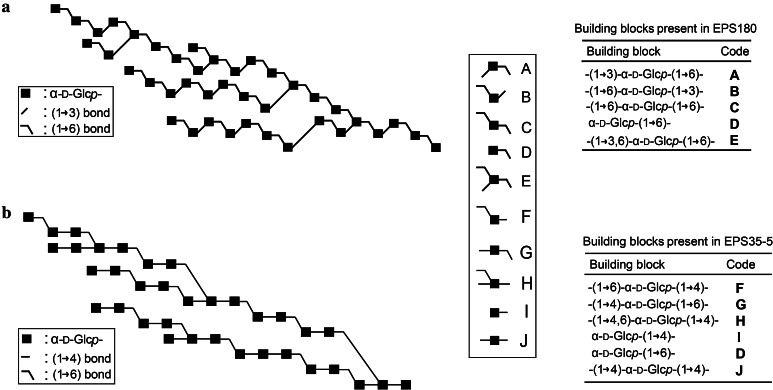
Visual representation of the composite models of the α-glucan polysaccharides produced by GTF180 (**a**) [82] and by GTFA (**b**) [59]

An intriguing question is how α-glucan synthesis by GSs is initiated, starting from sucrose. In other words, how does the α-glucan chain grow during the synthesis process? The structures of the GTFA and GTF180 α-glucan polysaccharides have been examined by 1D and 2D NMR spectroscopy, together with methylation analysis. Additionally, structural analysis has been performed on isolated oligosaccharides obtained by enzymatic hydrolysis, partial acid hydrolysis and Smith degradation of polysaccharides [59, 81–83]. Composite models of the α-glucans produced by wild-type and mutant GSs were constructed by combining all the information derived from the above-mentioned analysis (Fig. 6). These models provide valuable information about the structures of these α-glucans and the structure–function relationships of GTFA and GTF180 enzymes. Since GSs are able to transfer only one glucose unit in each reaction cycle, it is expected that α-glucans are synthesized by step-wise addition of one glucosyl unit to the growing glucan chains [13, 71]. Characterization of the oligosaccharides formed in the early phase of the reaction in time, therefore, would provide important information about the synthesis process. For that purpose, oligosaccharides formed by GTFA during the initial phase of the reaction were isolated and structurally characterized [84]. The results showed that the main oligosaccharides were sucrose elongated with alternating (α1 → 4) and (α1 → 6) linkages [84]. The abundance of these linkages is also shown in the composite model of the α-glucan synthesized by GTFA (Fig. 6). This demonstrated that polysaccharide synthesis starts with the transfer of glucosyl units to the non-reducing glucose end of sucrose with the formation of alternating (α1 → 4) and (α1 → 6) linkages. Subsequently, once the oligosaccharides reach a certain degree of polymerization (DP), branch points are formed and are further elongated with alternating (α1 → 4) and (α1 → 6) linkages. As a result, a branched α-glucan with large amounts of alternating (α1 → 4) and (α1 → 6) linkages is synthesized. This study provides direct evidence that sucrose is used as a primer by GTFA in the synthesis of α-glucan. The low-molecular mass (DP 20-30) and linear dextran produced by GTF-S3 from *S. sobrinus* were also found to be terminated with a sucrose moiety [85]. Moulis et al. also reported that DSRS of *L. mesenteroides* NRRL B-512F and alternansucrase of *L. mesenteroides* NRRL B-1355 used sucrose as an initiator of polysaccharide synthesis [65]. However, DSRS of *L. mesenteroides* NRRL B-512F also formed a series of isomalto-oligosaccharides (acceptor products starting from glucose) reaching DP higher than 25, while oligosaccharides with sucrose at the end did not exceed a DP higher than 12 [65]. In this work, both sucrose and glucose were proposed as initial acceptor for polysaccharide synthesis, but the latter was preferred [65]. However, whether this could be extended to other GSs is not known yet.

### GH70 GSs mode of action: processive versus non-processive

**Fig. 7:**
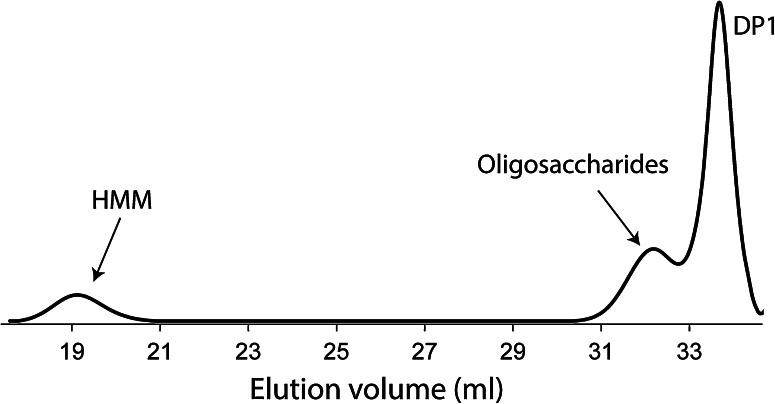
Molecular mass distribution of the products generated by incubating GTF180-ΔN (1.0 U/ml) with 0.1 M sucrose at 37 °C and pH 4.5. The HPSEC profile corresponds to the product mixture obtained after sucrose depletion. *HMM* high-molecular-mass polysaccharides, *DP1* monosaccharides (mainly fructose). This figure has been adapted from [99]

There has been a controversy about the mode of glucan chain elongation (processive versus non-processive). Previously, GH70 GSs were found to synthesize high-molecular weight polysaccharides during the early phase of reaction without the detection of intermediate oligosaccharides [86]. Consequently, GH70 GSs were assumed to act processively in the synthesis of α-glucan polysaccharides. Using a more sensitive method (high-performance anion-exchange chromatography equipped with an ED40 pulsed amperometric detection system, HPAEC-PAD), oligosaccharides were detected later on, which implied that GH70 GSs act non-processively as well [65]. Size-exclusion chromatography analysis showed that product mixtures formed by incubation of sucrose with GS enzymes generally contain high-molecular-mass (HMM) glucan and low-molecular-mass (LMM) oligosaccharides (Fig. 7) [65]; no intermediate size α-glucan products were detected. Kinetic analysis of polysaccharide synthesis by DSRS of *L. mesenteroides* NRRL B-512F revealed that HMM dextrans reached the maximum size after only 23 % of sucrose consumption with the simultaneous detection of oligosaccharides [65]. The polysaccharide synthesized by GTFA was also found to reach its maximum size in a relatively short time, with the simultaneous detection of oligosaccharides [84]. The reuteran polysaccharide size did not increase further, not even with the availability of excess sucrose [87]. The detection of HMM polysaccharide with a maximum size indicates a processive mode, while the detection of oligosaccharides points at non-processive mode. Therefore, taking into account all the information, Moulis et al. proposed a semi-processive mechanism of polymerization for GH70 GSs [65]. In the initial phase of the reaction, GH70 GSs catalyze the synthesis of oligosaccharides in a non-processive mode; when the oligosaccharides reach a certain size, polysaccharide synthesis proceeds in a processive mode. The structural basis for processive polysaccharide synthesis and non-processive oligosaccharide synthesis is proposed to be located in the repeat units in the C- and N-termini, which have been shown to be involved in glucan binding [56, 67, 88]. Mutants of DSRS with truncated A repeats (WYYFNXDGQAATGLQTIDGQTVFDDNGXQVG) were less efficient in polysaccharide synthesis compared with the wild-type enzyme [65]. This suggests that these repeats facilitate polysaccharide synthesis by anchoring the growing polysaccharide chain close to the active site. With the availability of the crystal structures, it became clear that the sequence repeats present in domain V formed a modular β-solenoid fold and domain V was proposed to play a role in carbohydrate binding [71, 72]. Indeed, truncation of domain V from GTF180-ΔN heavily impaired polysaccharide synthesis and increased the oligosaccharide synthesis [80]. Interestingly, mutation of residues around the acceptor binding sites partially restored the polysaccharide synthesis of GTF180-ΔNΔV [80]. Furthermore, several mutants of GSs, targeting amino acid residues close to the acceptor binding site, have been reported to produce different ratios of polysaccharide, oligosaccharide and glucose from sucrose [65, 89–92]. For instance, S628 (a residue C-terminal to the transition state stabilizer) mutants of GTFR (S628D and S628R) from *S. oralis* abolished polysaccharide synthesis and only produced short chain oligosaccharides [91]. Moulis et al. also reported that mutations of the residues C-terminal to the transition state stabilizer of DSRS from *L. mesenteroides* NRRL B-512F and alternansucrase from *L. mesenteroides* NRRL B-1355 abolished or reduced polysaccharide synthesis [65]. The polysaccharide synthesis increased significantly (about 2 fold) by mutation of residues around the acceptor substrate binding site (L940) of GTF180-ΔN [92]. These residues locate at acceptor substrate binding site and mutations at these subsites may affect the affinity of the enzyme with the growing glucan chain, resulting in decrease or increase of polysaccharide synthesis. These results suggest that the structural basis for processive polysaccharide synthesis lies in both domain V, and the acceptor substrate binding sites, representing remote and close binding sites for glucan chains, respectively. The glucan chain may also bind at the protein surface located in between these sites. Elucidation of the crystal structures of GH70 GSs with bound larger size glucan chains in future studies may reveal more information about such intermediate sites. However, previous attempts have so far been unsuccessful, indicating that obtaining such crystal structures is not trivial and may require other strategies.

It has also been shown that the reaction conditions, especially sucrose concentration, have significant effect on the product distribution of GSs. Kim et al. showed that the amount of HMM dextran produced by dextransucrase from *L. mesenteroides* B-512FMCM decreased while the amount of LMM dextran increased with increasing sucrose concentrations [93]. Dextrans of different molecular mass were synthesized at controlled sucrose concentrations, enzyme concentrations and reaction temperatures by dextransucrase from *L. mesenteroides* B-512FMC [94]. The ratio of oligosaccharide synthesis versus polysaccharide synthesis of GTFA from *L. reuteri* 121 is directly proportional to the concentration of sucrose. However, the sizes of the polysaccharides produced at different sucrose concentrations were identical [87]. At present, it remains unknown what determines the molecular size of the α-glucan polysaccharides synthesized by GS enzymes.

### Formation of branches

**Fig. 8:**
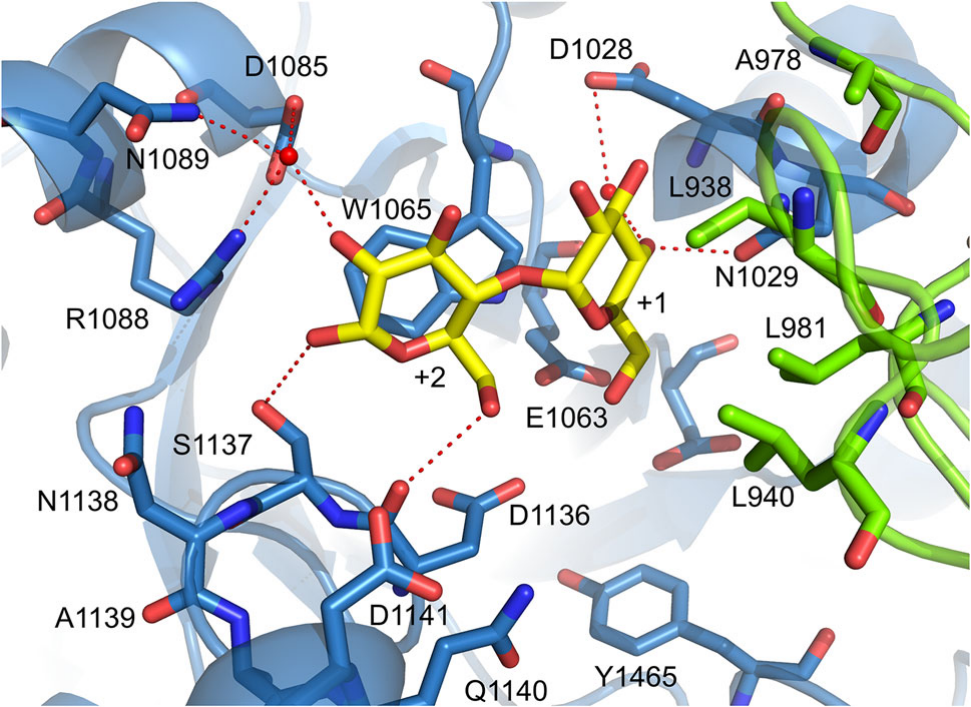
Maltose binding sites +1 and +2 in the crystal structure of the GTF180-ΔN maltose complex (PDB: 3KLL) [71]. Maltose is shown with yellow carbon atoms. Residues from domain A (*blue*) and B (*green*) surrounding the +1 and +2 subsites are indicated. Hydrogen bonds are shown as *dashed lines*

Most of the α-glucans synthesized by GSs are branched, to varying degrees [13, 15, 16]. Most GSs do not require an extra enzyme for the formation of branches in their α-glucan products. The mechanism for forming branch points remains unclear. Robyt and Taniguchi proposed that the formation of branched glucosyl units is through the acceptor reaction of GSs [95]. Site-directed mutagenesis has identified several amino acid residues that are involved in the formation of branched glucosyl units [96]. Mutation of three residues (S1137:N1138:A1139) following the transition state stabilizer residue (D1136) in GTF180 resulted in several mutants which synthesized α-glucans with a higher degree of branches [81]. These residues are located close to the +2 glucosyl unit of maltose in the crystal structure of GTF180-∆N in complex with maltose (Fig. 8) [71]. Residues D1085, R1088 and N1089 from α-helix 4 are located at the other side of the +2 glucosyl unit of maltose and they all make an indirect hydrogen bond with the +2 glucosyl unit of maltose through the same water molecule (Fig. 8). Multiple and single mutations in these residues resulted in mutants producing hyper-branched α-glucans (15–22 % branching) compared to that of wild-type (13 %) [97]. Single mutation studies showed that D1085 and R1088, but not N1089, are responsible for the increase in branched glucosyl units. Irague et al. also reported that mutations in the corresponding residues of DSRS of *L. mesenteroides* NRRL B-512F (D460, H463 and T464) increased the proportion of (α1 → 3) linkages [98]. Mutational studies of A978 and D1028 in GTF180 identified the involvement of a groove preceding the +1 subsite in the formation of branched glucosyl units [99]. Partially blocking the groove by mutating these residues to amino acid residues with larger side chains reduced the amount of branching linkages in the α-glucans synthesized. To summarize, the proportion of branched points in the α-glucans produced by GSs could be manipulated by mutations close to the acceptor substrate binding subsites. Thus, GS enzymes indeed may form branching linkages through the acceptor reaction, involving dissociation of the growing glucan chain from the acceptor binding subsites and subsequent rebinding in a different way that allows the formation of branch points. However, the question, when and where branching linkages are formed, remains unanswered.

In addition, (α1 → 2) branching GH70 sucrases have been reported [28, 35, 37]. With only sucrose as substrate, they are unable to polymerize glucosyl units and mainly catalyze the hydrolysis of sucrose and the formation of leucrose [28, 35, 37]. However, using sucrose as donor substrate and dextran as acceptor substrate, they catalyze the synthesis of (α1 → 2) branching linkages onto the dextran acceptor substrate [28, 35, 37]. Interestingly, (α1 → 3) branching GH70 sucrases have also been reported recently [100]. Structure–function relationship studies of these branching sucrases will provide valuable information about the mechanism of branching linkage formation.

## Linkage specificity determination of family GH70 GS enzymes

Although the active site of GSs is highly conserved, they produce α-glucans with different structures especially regarding glycosidic linkages [13, 16, 96]. These various α-glucans possess different physico-chemical properties such as molecular mass, solubility and viscosity. For instance, the solubility of mutan containing mainly (α1 → 3) linkages is generally low, while dextran with predominantly (α1 → 6) linkages is more soluble. Due to their ability to produce a diverse range of α-glucans, GSs have attracted interest for industrial applications in food, medicine, cosmetics, etc. [7, 34]. Dextran produced by the DSRS from *L. mesenteroides* NRRL B-512F is extensively applied as gelling, viscosifying and emulsifying agent in the food industry. Bakery products with dextran have improved softness and increased volume [101]. Dextran is also applied as size-exclusion chromatography material in research, and as a plasma expander in medicine [20, 102, 103]. Various α-glucans have been shown to possess anti-corrosion activity possibly by forming biofilms on the surface of steel [55, 104, 105]. Moreover, α-glucans and oligosaccharides formed by GSs have potential prebiotic activities and, therefore, can be used to stimulate growth of beneficial intestinal bacteria such as *Bifidobacterium* and *Lactobacillus* [106]. Linear and (α1 → 2)-branched dextran produced by the crude GS enzyme preparation from *L. citreum* NRRL B-1299 were reported to increase *Bifidobacterium* populations in vitro [107]. Monsan et al. reported that the addition of α-glucan to animal feed improved the weight gain of pig and broilers [108]. Therefore, the large variety of α-glucans and oligosaccharides with different structures holds great potential for industrial applications. A more detailed understanding of the linkage specificity of GSs may allow production of tailor-made α-glucans with desired properties.

The linkage specificity of GS enzymes appears to be determined by only a small number of amino acids [56, 90, 96]. As discussed above, all GSs initiate the reaction cycle by formation of a covalent C1-linked glucosyl-enzyme intermediate [15]. Consequently, it has been proposed that it is the way in which acceptor substrates bind at acceptor binding subsites that determine the linkage specificity of a GS enzyme, revealing the importance of amino acid residues at these sites [56, 65, 90, 96]. Prior to the availability of crystal structures, putative regions involved in acceptor binding site have been identified by alignment with family GH13 enzymes in view of their high sequence similarity [65, 89, 90]. These include residues C-terminal to the catalytic nucleophile D1025 (GTF180 numbering, conserved sequence motif II, Fig. 4), residues C-terminal to the acid base catalyst E1063 (motif III, Fig. 4) and residues C-terminal to the transition state stabilizer D1136 (motif IV, Fig. 4). These motifs display amino acid variations in different GSs [15]. Indeed, mutagenesis studies targeting these residues confirmed their importance for the product specificity of GSs. GTFA mutants P1026 V and I1029 V (motif II, GTFA numbering) displayed different product spectra with sucrose as substrate yielding higher levels of isomaltose and leucrose production [90]. Mutants A1066 N and H1065S:A1066S of GTFA (region III, GTFA numbering) produced similar products as wild type, but displayed lower activity [90]. In several GH70 GSs, residues located C-terminal to the catalytic transition state stabilizer (N1134:N1135:S1136: Q1137:D1138, motif IV, GTFA numbering) have been found to be critical for linkage specificity [65, 81, 90, 91]. Combined mutations in the tripeptide (N1134:N1135:S1136, motif IV, GTFA numbering) following the transition state stabilizing residue (D1133) in GTFA from *L. reuteri* 121 shifted GTFA linkage specificity from mainly (α1 → 4) linkages to (α1 → 6) linkages, indicating their involvement in acceptor substrate binding and hence linkage specificity determination [90]. Further mutation studies showed that, among these three residues, residue N1134 plays a major role in linkage specificity determination [89]. Similarly, mutations in the corresponding tripeptide in GTFR from *S. sobris* [91], GTF180 from *L. reuteri* 180 [81], and DSRS from *L. mesenteroides* NRRL B-512F [65] altered their linkage specificity as well. Mutation of the residues near the transition state stabilizer in GTFR (R628G:V630I:D717A) resulted in an increase of (α1 → 3) linkages in the polysaccharide produced [91]. Surprisingly, combining the mutations in motifs II and IV in GTF180, mutant V1027P:S1137 N:A1139S introduced 12 % (α1 → 4) linkages (not present in the wild-type) in the α-glucan produced [83]. Mutants of the fourth residue located at C-terminal transition state stabilizer of GTF180 (Q1140A and Q1140H) produced α-glucans with a higher percentage of (α1 → 6) linkages. The GTF180 Q1140E mutant also produced α-glucan with 3 % (α1 → 4) linkages [81]. In addition, the fifth residue following the C-terminal transition state stabilizer was also shown to be involved in linkage specificity determination. Random mutagenesis of D569 in GTF-I of *S. downei* showed that mutations at this position affected the structure of the α-glucan and the size of the synthesized oligosaccharides [109]. Mutations of the fifth residue following the transition state stabilizer in DSRI of *L. mesenteroides* NRRL B-1118 also affected the linkage composition of the products, with mostly increased (α1 → 3) linkages [110]. These results demonstrate that the involvement of residues located C-terminal to the transition state stabilizing residue in acceptor substrate binding is a general feature of GH70 GSs.

**Fig. 9:**
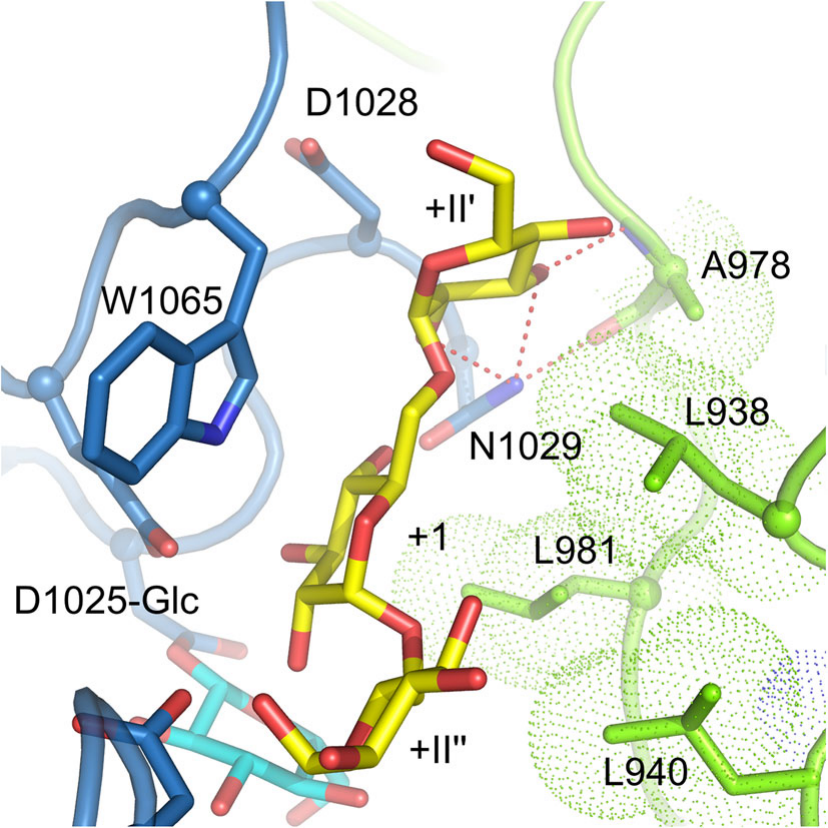
View of a docked isomaltotriose (*yellow* carbon atoms) in the active site of a modeled GTF180-ΔN glucosyl-enzyme intermediate (*cyan* atoms) [71], at the interface of domain A (*blue*) and B (*green*). The trisaccharide occupies subsites +1, +II′ and +II″; the latter two subsites are different from subsite +2 observed in the maltose complex

Elucidation of the crystal structure of GTF180-ΔN with a bound maltose acceptor substrate provided structural explanations for the effects of mutations observed previously [36, 71, 72] and confirmed that the amino acid residues at the acceptor binding site are critical for linkage specificity. Four bound maltose molecules (M1, M2, M3 and M4) were revealed by soaking GTF180-ΔN crystals with maltose [71]. However, the residues forming the binding sites for M2, M3 and M4 are not conserved within family GH70 GS enzymes and only have nonspecific interactions with GTF180-ΔN amino acid residues. Maltose M1 binds at the acceptor binding sites +1 and +2 with its C6 hydroxyl group of non-reducing end glucosyl moiety pointing towards the sucrose binding pocket. This C6 hydroxyl group activated by the acid/base catalyst (E1063) attacks the C1 of the glucosyl-enzyme intermediate, resulting in the formation of an (α1 → 6) linkage (Fig. 8). Thus, the binding mode of M1 explains how an (α1 → 6) linkage is formed with maltose as acceptor substrate (Fig. 8). At subsite +1, N1029 makes direct and indirect hydrogen bonds with the +1 C4 and C3 hydroxyl group, respectively; D1028 forms an indirect hydrogen bond with the +1 C4 hydroxyl group; residues from domain B (L938, L940, A978 and L981) shape the groove near the +1 subsite. These residues have rarely been targeted for mutagenesis studies prior to the availability of crystal structures [96]. In recent site-directed mutagenesis studies, these residues, especially residues from domain B, were found to be critical for linkage specificity and to display different roles [92, 99]. The L940 mutants synthesized α-glucans with larger amounts of (α1 → 6) linkages [92]. Surprisingly, the L940 W mutant produced linear α-glucan with only (α1 → 6) linkages; the synthesis of (α1 → 3) linkages was abolished completely. Docking studies with isomaltotriose showed that this tryptophan blocks a groove, preventing the reducing end of isomaltotriose to occupy the space observed in the wild-type [92]. Consequently, the C3 hydroxyl group of the non-reducing end glucose unit is too far away to attack the C1 of the glucosyl-enzyme intermediate. Instead, the C6 hydroxyl group is within the distance for forming (α1 → 6) linkages. This highlights the critical importance of the groove, where L940 is located, for (α1 → 3) linkage synthesis in GTF180. Residue A978 was found to be involved in branch points formation. Mutations of A978 to residues with a larger side chain (Leu, Pro, Phe and Tyr) reduced the branch formation in the α-glucans produced, while mutations to small residues (Gly and Ser) had no significant effects [99]. All D1028 mutants had increased amounts of (α1 → 6) linkages in their α-glucan products. The branching linkages were also influenced by D1028 mutations. Again, mutations to residues with a large side chain (Tyr and Trp) reduced the number of branches [99]. Docking studies showed that both A978 and D1028 are involved in shaping the groove above the +1 subsite, the space of which is required for acceptor substrate binding for formation of branching linkages (Fig. 9). Mutation of these two residues to bulky residues apparently partially blocked the groove, resulting in a decrease in branch point formation. L938 mutants were also shown to produce α-glucan with mostly increased (α1 → 6) linkages and thus also involved in linkage specificity. Mutations in N1029 resulted in an increase of (α1 → 3) linkages in the α-glucan products [99]. At acceptor binding site +2, residues S1137, N1138, A1139, Q1140, D1141, which have been shown to be important for linkage specificity, are located at one side of the +2 glucosyl unit (Fig. 8). It is worth to note that only S1137 has a direct hydrogen bond with the +2 C1 hydroxyl group. Indeed, S1137 was found to be the main determinant for linkage specificity in previous mutation studies [65, 81, 89–91]. This confirms the predicted involvement of these residues at this acceptor binding site. At the other side of the +2 glucosyl unit, residues D1085, R1088 and N1089 all possess an indirect hydrogen bond with the +2 C2 hydroxyl group through the same water molecule (Fig. 8). Single and combinatorial mutations studies showed that these residues are also involved in linkage specificity determination [97]. Mutations at these three residues, especially D1085 and R1088, introduced extra (α1 → 4) linkages in the α-glucans produced. Remarkably, some mutants also produce hyper-branched α-glucans with 15–22 % branched glucosyl units. Single mutational studies showed that residues D1085 and R1088, but not N1089, were responsible for the increase in branching linkages. Another combinatorial mutagenesis study, targeting the same residues in DSRS, also demonstrated their importance [98]. In addition, residue W1065 has hydrophobic stacking interactions with both the +1 and +2 glucosyl moiety of maltose [71]. In the complex of GTF180-∆N D1025 N with sucrose, this residue also has a direct hydrogen bond with the C1 hydroxyl group of the fructosyl moiety [71]. Mutating W491 of GTFI from *Streptococcus mutans* (W1065 in GTF180-∆N) to either glycine or alanine resulted in an enzyme devoid of detectable activity [111], demonstrating its essential role in the catalytic activities. Similarly, mutating W1065 of GTF180-∆N to non-aromatic amino acid residues heavily impaired the enzyme activity and abolished the synthesis polysaccharides (unpublished results). Mutant W1065F still retained the ability to synthesize polysaccharides with altered linkage composition. By now, only acceptor binding sites +1 and +2 have been mapped out and no crystal structure of a GH70 GS proteins with a bound higher molecular mass acceptor substrate is available; it remains unclear whether amino acid residues at further acceptor binding sites are determinants for linkage specificity. These remote acceptor binding sites are difficult to identify by sequence alignment or to predict based on a crystal structure, because they may not be conserved.

In some cases, it has been shown that the next linkage type is also determined by the previous linkage formed. One example is the alternansucrase (ASR) from *L. mesenteroides* NRRL B-1355, which catalyzes the synthesis of alternan with alternating (α1 → 6) and (α1 → 3) linkages. This suggests that an acceptor substrate with an (α1 → 6) linkage between the +1 and +2 subsites favors the formation of an (α1 → 3) linkage in the next reaction cycle; in turn, an (α1 → 3) linkage at this position induces the formation of an (α1 → 6) linkage. It has been proposed that the non-reducing end of the acceptor substrate is not well stabilized at the +1 subsite, while the glucosyl residue at the +2 site of ASR is more stabilized due to the stacking interaction with W675 and/or Y768 (Fig. 4) [65]. Residue Y768 is unique for ASR and has been proposed to provide a 2nd stacking platform for a glucosyl residue at the +2 subsite. Thus, the linkage between the +1 and +2 glucosyl residues of a bound acceptor substrate probably determines that the +2 glucosyl residue stacks with either residue W675 or Y768, resulting in the accessibility of the C3 or C6 hydroxyl group of the non-reducing end glucosyl residue to the glucosyl-enzyme intermediate. Mutant Y768S:D769E:A770 V of the alternansucrase of *L. mesenteroides* NRRL B-1355 was unable to synthesize alternan and produced more oligo-dextrans from sucrose and maltose [65]. Another example is GTFA from *L. reuteri* 121 [59]; characterization of the oligosaccharides initially produced by GTFA revealed that the most prominent products were oligosaccharides with alternating (α1 → 6) and (α1 → 4) linkages [84]. However, GTFO from *L. reuteri* ATCC 55730, which shares high similarity with GTFA, synthesizes a reuteran with a high amount of (α1 → 4) linkages instead of alternating (α1 → 6) and (α1 → 4) linkages. The determinants of the different linkage specificity of GTFA and GTFO are currently under investigation. GTF180 from *L. reuteri* 180 synthesizes an α-glucan with (α1 → 6) and (α1 → 3) linkages [57]. Structural analysis of this α-glucan showed that it is built up with different lengths of isomalto-oligosaccharides, interconnected by single (α1 → 3) linkages [82]. All (-)α-D-Glc*p*-(1 → 3)- units were found to be 6-substituted and no consecutive (α1 → 3) linkages were found [82]. Taken together, these examples show that the linkage specificity of GH70 GSs is also depending on the linkage present between the +1 and +2 acceptor substrate binding site. However, unlike the situation in ASR, no 2nd aromatic residue was found at subsite +2 in GTFA and GTF180; multiple mechanisms of alternating linkage formation thus occur.

To conclude, these mutagenesis studies showed that the linkage specificity of GH70 GSs is determined by an interplay of different amino acid residues from both domains A and B, shaping the acceptor binding sites. The specific interactions between the acceptor substrate and its acceptor binding sites determine which hydroxyl group of the non-reducing end glucosyl moiety of an acceptor substrate is capable of attacking the glucosyl-enzyme intermediate to form the next α-glycosidic linkage. Therefore, it is not one but several amino acid residues of GH70 GSs that determine their enzyme linkage specificity. This explains why, even with different amino acid residues at a certain position, different GSs still may have similar linkage specificities; and with the same amino acid residues at a certain position, they catalyze the synthesis of different linkages. Although the contribution of different residues complicates the rationalization of specificity in GSs, 3D structure-guided mutagenesis is an effective approach for changing the linkage specificity of GS enzymes and producing novel α-glucans. Together, the various mutagenesis studies, supported by 3D structural observations, contribute to a better understanding of the GH70 GS linkage specificity.

## Biochemical properties of GTFB- and GTFC-like 4,6 α-glucanotransferase enzymes

Although sucrose remains the canonical D-glucosyl donor substrate for GSs, some GS enzymes also can use oligosaccharides as donor/acceptor substrates [112]. In this so-called disproportionation reaction, the glucosyl group is transferred from one saccharide donor to an identical or similar saccharide acceptor substrate [112]. Binder et al. found that dextransucrase from *L. mesenteroides* NRRL B-512F and GTF-S from *S. mutans* 6715 have disproportionation activity with isomalto-oligosaccharides, malto-oligosaccharides and panose.

## GTFB-like 4,6 α-glucanotransferase enzymes

This disproportionation activity of GSs has been overlooked for decades due to its relatively low activity compared to the glucosyl transfer from sucrose. However, it was found that *Lactobacillus reuteri* 121 possessed a GS-like enzyme which was inactive on sucrose, and instead used malto-oligosaccharides as donor and acceptor substrates [18]. The gene encoding this enzyme designated as GTFB, is located upstream the gene encoding the GTFA that catalyses the synthesis of reuteran with (α1 → 4) and (α1 → 6) linkages from sucrose [58]. GTFB predominantly cleaves an (α1 → 4) glycosidic linkage from the non-reducing end of the donor substrate [(α1 → 4)-glucan] and transfers the cleaved glucosyl unit to the non-reducing end of another (α1 → 4)-glucan acceptor substrate, forming mainly (α1 → 6) linkages. Products formed with an (α1 → 6) linkage at the non-reducing end become better acceptor substrates and are further elongated in a linear manner with (α1 → 6) linked glucosyl units. This results in the formation of isomalto/malto-oligosaccharide and polysaccharide mixtures with increasing percentages of (α1 → 6) linkages [113, 114]. Two more GTFB-like enzymes (GTFW from *L. reuteri* DSM 20016 and GTFML4 from *L. reuteri* ML1) were characterized later on [18, 114]. In view of their clearly distinct reaction specificity, these enzymes have been designated as 4,6-α-glucanotransferases (4,6-α-GTs) (EC 2.4.1.-). Besides these 3 biochemically characterized GTFB-like 4,6-α-GTs (GTFB, GTFW and GTFML4), 46 putative GTFB-type 4,6-α-GTs are currently found in the GenBank database. Except for three from *Pediococcus* strains, they are all found within the genus *Lactobacillus*.

Interestingly, GTFB converts amylose and high amylose starches into isomalto-/malto-polysaccharides (IMMP) with high percentages of (α1 → 6) linkages [115]. For example, incubation of the amylose V of potato with GTFB yielded a linear product with 91 % of (α1 → 6) linkages. The degree of branching in starch substrates negatively correlates with the amount of (α1 → 6) linkages in the produced IMMP; it was, therefore, postulated that the GTFB enzyme cannot get across the branch points and, thus, cannot act on internal (α1 → 4) linkages [115]. This was further confirmed by the fact that when using debranched starches as substrate, GTFB introduced a higher percentage of (α1 → 6) linkages in the IMMP product [115]. Structural and size analysis showed that the IMMP constituted an entirely novel class of α-glucans, e.g., different from the known dextrans and isomalto-oligosaccharides. These IMMP are proven soluble dietary fibers since the segments rich in (α1 → 6) linkages largely pass the human small intestine and enter into the large intestine [115].

**Fig. 10:**
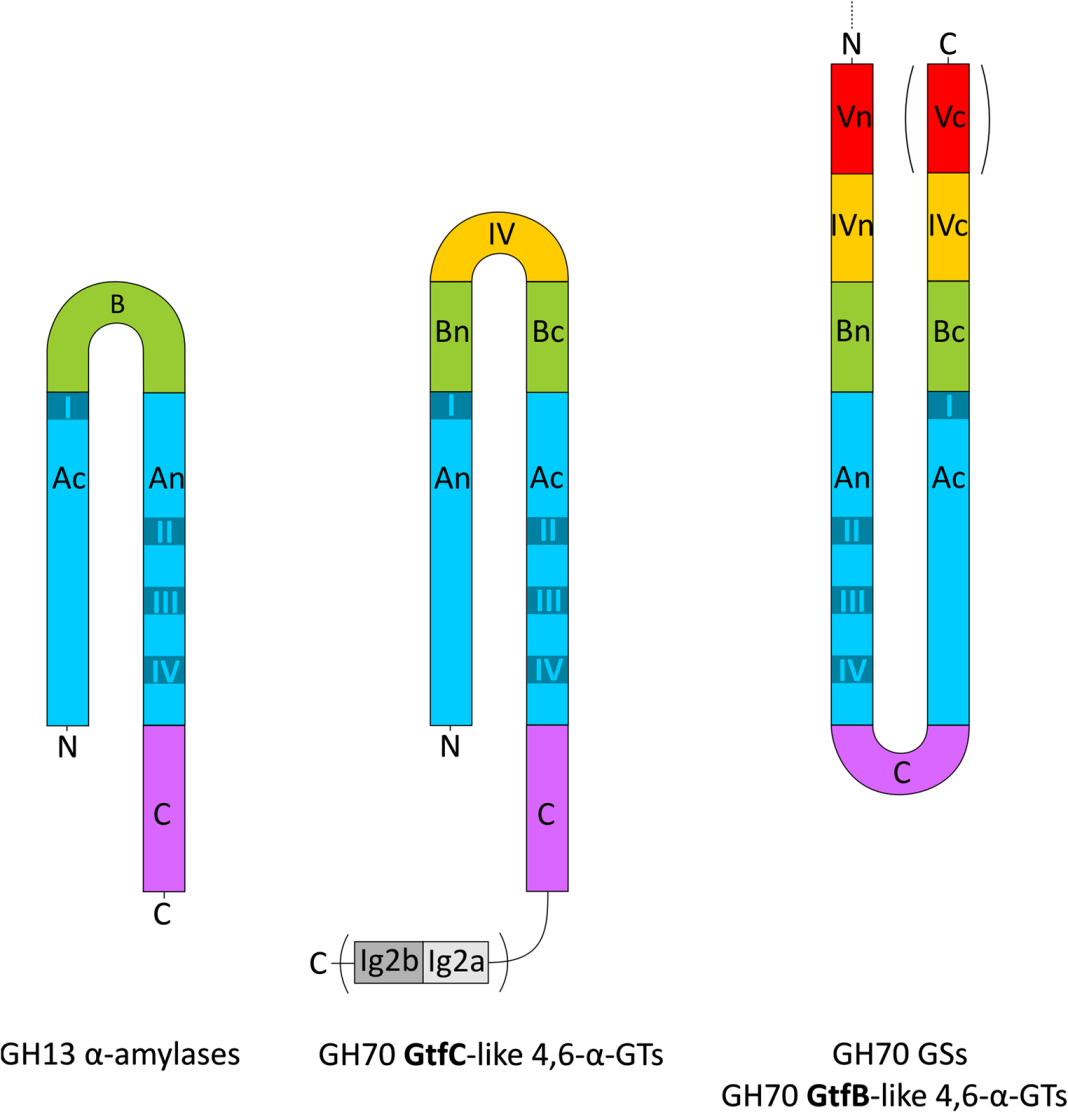
Schematic representation of the domain organization of GH13 α-amylases and GH70 4,6-α-glucanotransferases and GH70 GSs. Domains A, B, C, IV, and V are colored in *blue*, *green*, *magenta*, *yellow*, and *red*, respectively. Ig2-like domains are indicated in gray. Brackets indicate that the Ig2-like domains are not identified in all GtfC-like enzymes, and that the C-terminal half of domain V is not found in all GtfB-like 4,6-α-glucanotransferases [17]. This figure has been adapted from [17]

The GTFB-like 4,6-α-GT enzymes show about 50 % amino acid sequence identity with GH70 GSs and clearly belong to family GH70 [18, 114]. Primary structure analysis revealed that GTFB-like 4,6-α-GTs, like GH70 GSs, have the same domain organization in that domains A, B, C and IV are made up from discontinuous N- and C-terminal stretches of the polypeptide chain (Fig. 10). It should be noted that, while in most GH70 GSs domain V consists of both N- and C-terminal polypeptide segments, domain V of GTFB is smaller since it only consists of an N-terminal polypeptide segment. The catalytic domain of GTFB-like 4,6-α-GTs also consists of a circularly permutated (β/α)_8_ barrel with conserved sequence motifs I to IV as in family GH70 GS enzymes (Fig. 10). The six conserved residues in conserved sequence motifs I to IV including the three catalytic residues (the nucleophile D1025, the acid/base catalyst E1063 and the transition state stabilizer D1136, GTF180 numbering) in GH70 GSs and family GH13 enzymes are also present in GTFB-like 4,6-α-GTs (Fig. 4). Similar to the GH70 GSs, the seventh conserved residue (His134, α-amylase of *Bacillus licheniformis* numbering) of family GH13 enzymes is also replaced by a Gln residue in GTFB-like 4,6-α-GTs. Besides the similarities, a large number of amino acid residues in motifs I to IV of GTFB-like 4,6-α-GTs show variations as compared to GH70 GSs, especially for the residues contributing to the -1, +1, and +2 donor/acceptor binding subsites in motifs I to IV (Fig. 4). For example, amino acid residues downstream of the acid/base catalyst are completely different in GH70 GSs and GTFB-like 4,6-α-GTs (Fig. 4). Specifically, residue W1065 (GTF180 numbering) which is highly conserved in GH70 GSs (except for DSRECD2) is replaced by a Tyr residue in GTFB-like 4,6-α-GTs. Residues located C-terminal of the transition state stabilizing residue in conserved sequence motif IV, part of acceptor subsite +2 in GH70 GSs and critical for their linkage specificity, are also very different in GTFB-like 4,6-α-GTs. These differences in amino acid residues may contribute to the distinct linkage specificity of GTFB-like 4,6-α-GTs. Further mutational studies may allow elucidation of the functional roles of these amino acid residues in determining the substrate specificity and product linkage specificity of GTFB-like 4,6-α-GTs.

## GTFC-like 4,6-α-glucanotransferase enzymes

Recently, we have reported the identification of a second novel GH70 subfamily with 4,6-α-glucanotransferase activity on maltodextrins and starch (designated as GTFC-like 4,6-α-GTs), present in *Exiguobacterium* and *Bacillus* strains. Both *Exiguobacterium* and *Bacillus* are genera within the class Bacilli and members of the low GC phylum of Firmicutes, but they do not belong to the lactic acid bacteria. The *Exiguobacterium sibiricum* 255-15 GTFC was selected as the first representative member of this group and characterized in detail [17]. *E. sibiricum* 255-15 is a free-living, psychrotrophic, nonsporulating, Gram-positive bacterium that was isolated from a 3 million year old Siberian permafrost core [116, 117]. This bacterium was found to produce exopolysaccharides, proposed to be the result of the activity of a putative GH70 enzyme, later designated as GTFC, identified in its genome [116, 117]. Analysis of sequence-based relatives of the *E. sibiricum* GTFC, using BLAST, revealed that the closest homologs of these GTFC-like 4,6-α-GTs are GTFB-like proteins (with statistically significant E values), even though they share only 30 % of sequence identity. Similar to the GTFB-like 4,6-α-GTs, GTFC catalyzes cleavage of (α1 → 4) glycosidic linkages and synthesis of consecutive (α1 → 6) linkages. GTFC differs from GTFB in converting amylose/starch substrates into Isomalto-/malto-oligosaccharides (IMMO), instead of the (modified) polymers (IMMP) synthesized by GTFB [115]. The similar reactions catalyzed by these two enzyme types correlate well with the highly conserved motifs I to IV, particularly in residues forming the acceptor binding subsites (Fig. 4). Similar to GTFB-like proteins, a Tyr residue in GTFC-like 4,6-α-GTs proteins replaces the subsite +1/+2 Trp residue conserved in almost all GH70 GSs (W1065 in GTF180-ΔN). The amino acids following the putative transition state stabilizer residue in GTFC homologs are very different from those in GH70 GSs (Fig. 4). These amino acid residues clearly share higher similarity with GTFB-like 4,6-α-GTs. GTFB- and GTFC-like 4,6-α-GTs all have an one amino acid gap at position 1139 (GTF180 numbering) and share residues Gln, Lys and Asn at positions 1137, 1140 and 1141, respectively. However, at position 1138, GTFB-like 4,6-α-GTs all have an Arg residue, while in GTFC-like 4,6-α-GTs a Glu residue is present. Most importantly, GTFC-like 4,6-α-GTs differ from GTFB-like 4,6-α-GTs and GH70 GSs by a non-circular permutation of conserved sequence motifs I to IV (Fig. 10). In GTFC-like 4,6-α-GTs, the order of these conserved sequence motifs is identical to that in GH13 (I-II-III-IV). Thus, the domain organization of the catalytic core (domains A, B, and C) of these GTFC-like 4,6-α-GTs resembles that of GH13 α-amylases, lacking the circular permutation of the (β/α)_8_ barrel (Fig. 10). A striking feature of these GTFC-like 4,6-α-GTs, with respect to GH13 amylases, is that they possess an extra contiguous domain IV interrupting domain B (Fig. 10). Domain IV is exclusively found in family GH70 enzymes and thus far its function has remained unknown. In addition, several (but not all) GTFC-like proteins contain one or two Ig2-like domains of unknown function, whereas they lack the variable N-terminal domain and domain V typically present in GH70 members. In view of the clear sequence similarity between GTFC-like 4,6-α-GTs and other GH70 enyzmes, it was found more appropriate to classify these GTFC-like proteins as a new subfamily in family GH70 than to establish an entirely new family. They thus constitute the first subfamily GH70 enzymes that lack the circularly permutation of the (β/α)_8_ barrel that hitherto had been considered as a very characteristic difference between families GH13 and GH70 [17, 74].

## Evolutionary relationships between family GH70 and GH13 members

The GH13 enzymes, degrading or modifying mainly starch-like substrates, constitute one of the largest Glycoside Hydrolase family at present in the CAZy database (www.cazy.org) and are found in a very wide range of organisms from all kingdoms [118, 119]. α-Amylase enzymes, catalyzing hydrolysis of internal (α1 → 4) glycosidic bonds in starch and related substrates, are the main representatives of family GH13. Despite the fact that starch and sucrose acting GH13 and GH70 enzymes differ in their overall activities, there is no doubt that they share a common ancestor [74, 120]. Members of family GH13 and GH70 share a (β/α)_8_ barrel domain and employ a similar catalytic mechanism involving a covalent glucosyl intermediate and the retention of the α-configuration in their products [121]. Structurally, the catalytic core of GH70 GSs, composed of A, B and C domains, was found to be similar and can be superimposed on the A, B and C domains present in α-amylases [71]. Moreover, the GH13 and GH70 protein sequences exhibit 4–7 conserved sequence motifs that can be used as sequence fingerprints for the individual enzyme specificities [122].

GTFB-like 4,6-α-GTs constitute a first GH70 subfamily, unable to act on sucrose, but active on starch and maltodextrins, cleaving (α1 → 4) linkages and synthesizing consecutive (α1 → 6) linkages [18, 113, 114]. Considering the structural similarity of GTFB-like 4,6-α-GTs with GH70 GSs and their reaction similarity with family GH13 using (α1 → 4) glucans as substrates, GTFB-like 4,6-α-GTs were proposed to be an evolutionary intermediate of family GH13 α-amylases and GH70 GSs [18, 114]. The characterization of GTFC of *Exiguobacterium sibiricum* 255-15 showed that it has a similar activity as GTFB-like 4,6-α-GTs, but, like GH13 family enzymes, lacks a permutated (β/α)_8_ barrel.

**Fig. 11:**
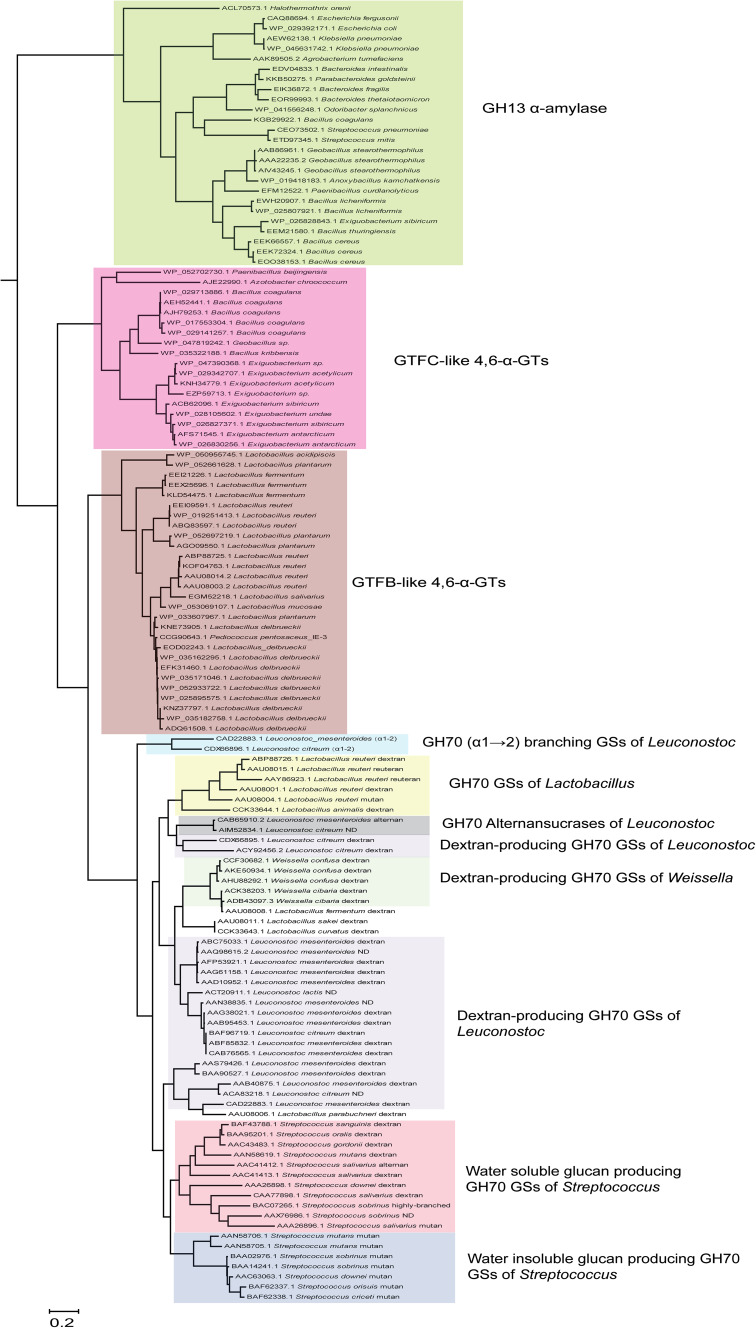
Phylogenetic tree analysis of GH70 and GH13 proteins. The evolutionary tree is based on the alignment of the complete sequences of all biochemically characterized GH70 GSs, (putative) GH70 GTFB-like and GTFC-like 4,6-α-GTs and (putative) GH13 α-amylases identified by a BLAST search using the *E. sibiricum* GTFC protein as query sequence. The evolutionary history was inferred using the Maximum Likelihood method based on the JTT matrix-based model. The *bar* represents a genetic distance of 0.2 substitution per position. Each sequence is labeled with its Genbank accession number and bacterial origin. In the case of the biochemically characterized GH70 GSs, their α-glucan polysaccharide products are also shown. Details of the aligned sequences are shown in Table S1

The BLASTp analysis of the *E. sibiricum* GTFC 4,6-α-GT revealed that the closest homologs of these GTFC type of proteins are GTFB-like 4,6-α-GTs, whereas the next hits were (putative) GSs followed by (putative) GH13 α-amylases. The evolutionary relationships between all the biochemically characterized GSs as well as the (putative) GH70 4,6-α-GTs and (putative) GH13 α-amylases identified by this BLASTp search are depicted in the phylogenetic tree constructed based on the alignment of the complete sequences (Fig. 11). GH70 proteins are divided into three clearly separated clusters in the phylogenetic tree: GH70 GSs, GTFB-like and GTFC-like 4,6-α-GTs. Phylogenetically, GTFC-like 4,6-α-GTs are more closely related to GTFB-like 4,6-α-GTs, but are positioned between family GH70 enzymes and GH13 α-amylases, a fact that is also reflected in their non-permuted GH13 type of domain architecture. The position of the GTFB-like 4,6-α-GTs, in between GH70 GSs and GTFC-like 4,6-α-GTs, may be related to their high sequence identity to GH70 GSs. Most of the biochemically characterized family GH70 GSs cluster in clades in the phylogenetic tree, reflecting their bacterial hosts, but with a few exceptions (Fig. 11). Most GSs of *Leuconostoc* produce dextran types [mainly (α1 → 6)] of polysaccharides and they form three separate clusters through the tree. The *Leuconostoc* alternansucrases and (α1 → 2) branching sucrases form separate clades from the main cluster of *Leuconostoc* GSs, reflecting their different product specificity. GSs from *Streptococcus* form two main clades, which produce soluble (dextran) and insoluble (mutan) polysaccharides, respectively. The characterized GSs of *Weissella* are closely related and produce dextran types of polysaccharides. Also *Lactobacillus* derived GSs cluster together; however, they produce different types of polysaccharides (reuteran, dextran and mutan). It is worth to note that within GH70 GSs, the (α1 → 2) branching GS is phylogenetically most close to GTFB-like 4,6-α-GTs. The reason for this is not clear.

**Fig. 12:**
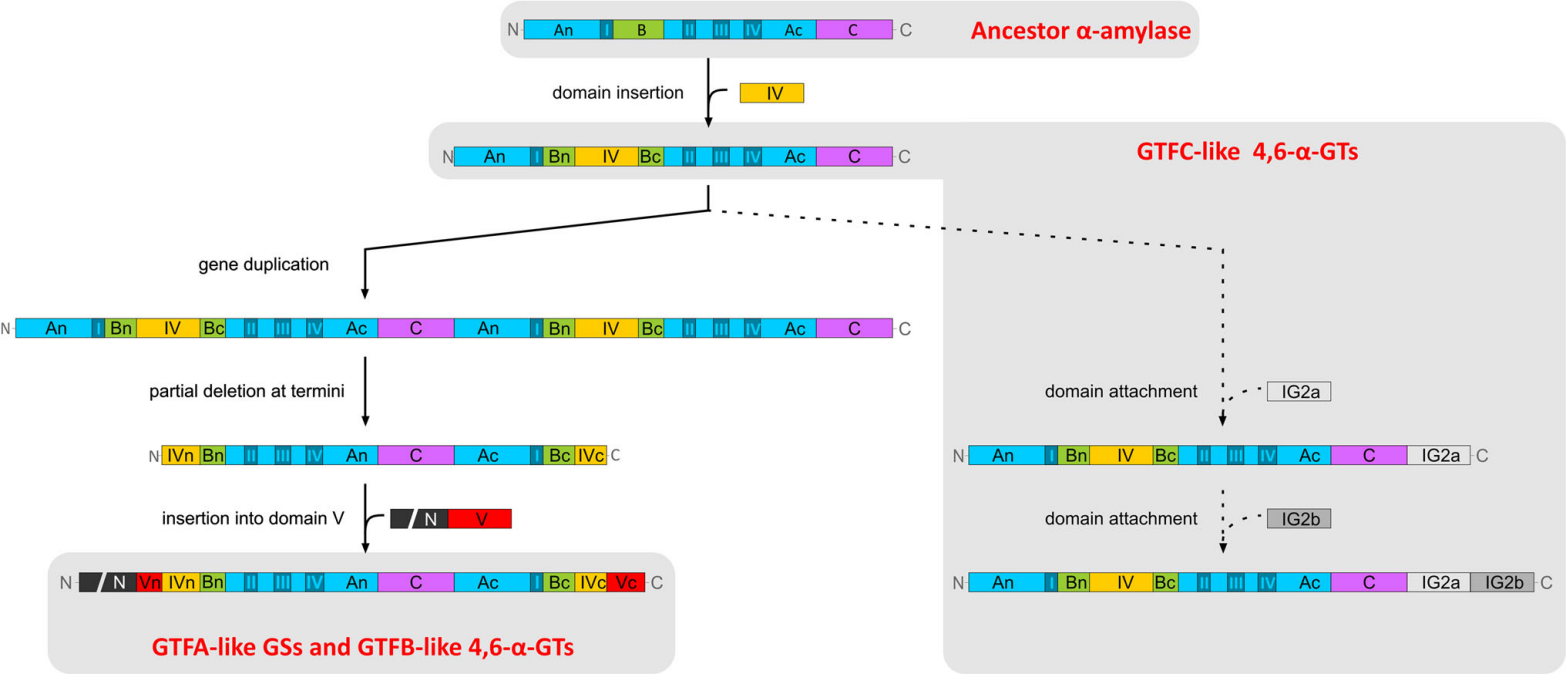
Proposed evolutionary pathway based on the “permutation per duplication model” leading to the unusual domain organization of GH70 GSs and GTFB-like 4,6-α-GTs. Insertion of domain IV into domain B of the ancestor α-amylase led to the formation of the “intermediate” GTFC subfamily. Formation of the GTFC “intermediate” would be followed by the circular permutation (gene duplication and partial terminal deletions), and domain insertion events leading to GH70 GSs and GTFB-like 4,6-α-GTs. Some of the GTFC-like 4,6-α-GTs members acquired Ig2-like domains (route indicated by dotted arrows). Sequence segments forming domains A, B, C, IV, and V are *colored* as before in *blue*, *green*, *magenta*, *yellow*, and *red*, respectively. Ig2-like domains are indicated in *gray*. This figure has been taken from [17]

It has been proposed that GH70 proteins evolved from a precursor GH13 α-amylase rather than vice versa, since the latter are widespread through different taxonomic groups and occur even in Archaea [18]. It appears more likely that amylolytic activity emerged first, and that later in time transglycosidase activity arose by optimization of the acceptor binding subsites that allowed the recognition and utilization of the products formed by the enzyme as acceptor substrates. The elucidation of the crystal structure of GTF180 GS prompted Vujičić et al., 2010 to propose that GSs emerged from an ancestor α-amylase by an evolutionary pathway based on the “permutation per duplication model” [123]. Based on this pathway, the circularly permuted domain A, discontiguous domain B, and contiguous domain C characteristic for GSs are the result of the duplication, fusion and partial truncation of an ancestor GH13 α-amylase gene. However, it remained unclear whether these sequences of gene rearrangements that resulted in the circular permutation occurred before or after the insertion of domain IV into the precursor gene. The existence of GTFC-like 4,6-α-GTs displaying an α-amylase GH13-like fold but with domain IV inserted in domain B sheds light on the evolutionary history of family GH70 proteins (Fig. 12). First, insertion of domain IV may have led to the “intermediate” GTFC subfamily. Later, the unusual “U” fold domain organization of GS and GTFB-like 4,6-α-GTs evolved from a GTFC-like 4,6-α-GTs intermediate via the permutation-by-duplication mechanism, followed by insertion into the gene encoding domains V and N. In parallel, some but not all of these GTFC-like 4,6-α-GTs acquired Ig2-like domains at their C-terminus. While the above pathway describes the evolutionary changes in domain organization of GH13 and GH70 enzymes, it should be noted that the reaction specificity of the respective enzymes also has changed from one (sub)family to another. As of today (November, 2015), we do not know the structural details related to these changes, since no crystal structures have been reported yet for GTFB- or GTFC-like 4,6-α-GTs.

## Future perspectives

The number of putative GH70 GSs is increasing with the number of genome sequences available. At present, GSs are exclusively found in LAB, mainly from *Leuconostoc*, *Lactobacillus*, *Streptococcus*, and *Weissella* [15, 96]. However, only a relatively small number of these GSs have been biochemically characterized [15]. Only a single GS-encoding gene has been identified in the genus *Oenococcus* (*Oenococcus oeni* PSU-1); this enzyme has not been characterized yet and at present it remains unknown whether it has special properties compared to GSs found in the other LAB [124]. It also has remained unclear why GSs are only found in LAB. This may reflect a special feature of LAB, for instance their strong association with surfaces, present in biofilms. The question remains whether the increasing genome sequence data also will result in identification of GSs in non-LAB.

The number of GTFB-like 4,6-α-GTs is also increasing with the availability of genome sequence data. Very recently, the genome sequences of 213 *Lactobacillus* strains and associated genera have been sequenced and analyzed through comparative genomics [125]. This genome sequencing initiative has contributed to further increase the number of available GTFB-like 4,6-α-GTs protein sequences. As of November 2015, the GTFB-like 4,6-α-GT subfamily contains 46 proteins. Most of these GTFB-like 4,6-α-GTs are found in *Lactobacillus* (except a 3 putative GH70 enzymes identified in *Pediococcus* strains). Only 3 GTFB-like 4,6-α-GTs have been biochemically characterized showing a similar substrate specificity and product linkage specificity [cleaving (α1 → 4) and synthesizing consecutive (α1 → 6) linkages] [18, 114]. GSs are capable of synthesizing all four possible types of α-glycosidic linkages in α-glucans [96]. The biochemical characterization of additional GTFB-like proteins may result in identification of novel enzymes, displaying different product linkage specificities. Such biochemical data will also contribute to a better understanding of the GTFB structure–function relationships. The same is valid for GTFC-like 4,6-α-GTs, which only have been found in different Gram-positive bacteria (i.e. *Exiguobacterium* and *Bacillus*). Only a single GTFC-like 4,6-α-GT has been biochemically characterized [17] and further cloning and biochemical characterization will provide more detailed insights into their substrate and product linkage specificity. GTFB-like 4,6-α-GTs and GTFC-like 4,6-α-GTs represent the evolutionary intermediates between family GH70 GSs and family GH13 enzymes. Further genome sequencing is likely to result in identification of novel putative GH13-GH70 intermediates representing additional GH70 subfamilies; their detailed biochemical characterization may provide detailed insights into the evolutionary events that have occurred.

Previous mutagenesis studies [65, 81, 89–91, 96, 109, 110, 126], elucidation of the crystal structures of GH70 GS enzymes synthesizing different glycosidic linkages [36, 71–73, 79] and recent site-directed mutagenesis studies based on these 3D structures [92, 98, 99] contribute to a better understanding of GH70 GS structure–function relationships, especially regarding linkage specificity. These studies showed that the linkage specificity of GH70 GSs is determined by the interplay of residues from both domains A and B, forming acceptor substrates binding subsites. By now, only acceptor substrate binding subsites +1 and +2 of maltose have been studied in detail [71]. The availability of three-dimensional GS structures with a bound longer carbohydrate substrate, and/or a bound branched oligosaccharide, would greatly support unraveling additional acceptor substrate binding sites. With the presently available 3D structural information, combinatorial mutagenesis (targeting several residues) allows diversification of the glycosidic linkage composition of the α-glucans produced. In a recent study of dextransucrase DSRS of *L. mesenteroides* NRRL B-512F, guided by the homologous GTF180-∆N crystal structure, several residues forming acceptor substrate binding sites were targeted for combinatorial mutagenesis [98, 127]. Characterization of the mutants obtained revealed synthesis of a range of novel α-glucans with an altered relative amount of (α1 → 3) linkages (3–20 %) [98, 127]. A directed evolution approach combined with a high-throughput screening facility for specific linkage types represents an alternative approach to study linkage specificity [128]. Such studies are likely capable of identifying hotspot regions that are located more distantly from the acceptor substrate binding sites, which may allow fine tuning of the acceptor substrate binding sites [129]. Biochemical and structural characterization of mutant GS enzymes and their products synthesized will generate a detailed understanding of the structure–function relationships of GS enzymes. Such detailed insights may allow rational construction of GS mutants with acceptor substrate binding subsites providing desired physico-chemical microenvironments that produce tailor-made α-glucans, i.e. with desired linkage type distributions, degree of branching, and molecular mass. Currently, the determinants of product size of GH70 GSs have remained largely unknown. An effective way to control the sizes of the α-glucans synthesized by GSs is required for future industrial applications. Also the (thermos)stability of GSs needs to be improved; the availability of protein crystal structures facilitates a computational design approach to achieve this [130].

The donor substrate specificity of GH70 GSs is limited to sucrose, resulting in the transfer of only a glucosyl moiety. Various sucrose analogs have been synthesized, in which the glucosyl unit of sucrose is replaced by alternative glycosyl moieties [131–133]. The sucrose analogs α-d-xylopyranosyl-β-d-fructofuranoside (Xyl-Fru), α-d-mannopyranosyl-β-d-fructofuranoside (Man-Fru), α-d-galactopyranosyl-β-d-fructofuranoside (Gal-Fru) and α-d-fucosylpyranosyl-β-d-fructofuranoside (Fuc-Fru) have been synthesized by transferring the fructosyl unit of sucrose to monosaccharides acceptor substrates using fructansucrase enzymes [133–136]. The use of sucrose analogs as donor substrates by GS enzymes would allow transfer of a wider range of monosaccharides for oligosaccharide and glycoconjugate synthesis. GSs will need to be engineered to improve their ability to transfer such different glycosyl moieties. In a recent study, GTFA of *L. reuteri* 121 was reported to use α-d-allopyranosyl-β-d-fructofuranoside as donor substrate and to transfer an allose unit to several acceptor substrates [137]. The use of sucrose analogs as donor substrates provides a promising opportunity to extend the glycodiversity of GS products.

GTFB-like 4,6-α-GTs show the same domain organization and share a circularly permutated (β/α)_8_ barrel with GH70 GSs [18, 114]; however, they show clearly different substrate specificity and product linkage specificity [18, 114]. Identification of the determinants for these characteristics is an interesting topic for future research. Elucidation of the 3D structure of GTFB-like 4,6-α-GTs and mutational studies will provide a firm basis for such studies. GTFC-like 4,6-α-GTs differ from GTFB-like 4,6-α-GTs and GH70 GSs by a non-permutated (β/α)_8_ barrel. Elucidation of the 3D structure of GTFC-like 4,6-α-GTs and mutational studies may also serve to elucidate features that determine their substrate and product linkage specificity. The crystal structures of GH70 GSs showed that these proteins follow a U-shape course to form different domains; the same is probably true for GTFB-like 4,6-α-GTs while the overall folding of GTFC-like 4,6-α-GTs may be similar to that of family GH13 enzymes and different from those of GSs and GTFB-like 4,6-α-GTs.

In recent years, this field has advanced by the characterization of novel α-glucans containing different (ratios of) glycosidic linkages and various degrees of branching, produced from sucrose by newly derived GH70 GS enzymes and their mutant derivatives, but also by the identification and characterization of GH70 subfamily GTFB-like 4,6-α-GTs and GTFC-like 4,6-α-GTs, using maltodextrin/starch as substrates. The physico-chemical properties of these α-glucans remain to be investigated to elucidate their structure–function relationships. Industrial application processes eventually may be developed based on the knowledge obtained in such studies.

In view of recent developments, characterization of new (sub)family GH70 enzymes, elucidation of their crystal structure, (rational) enzyme engineering and substrate engineering (sucrose analogs), the prospects of producing tailor-made α-glucans are promising. This further enhances the applicability of GH70 enzymes as biocatalysts, and their products in food and medicine, and in the cosmetic industry. Moreover, their structural and functional relationships with family GH13 enzymes provide a very interesting showcase for the analysis of protein evolution processes in nature.

### Electronic supplementary material

Below is the link to the electronic supplementary material.

Supplementary material 1 (DOC 179 kb)

## Abbreviations

EPS: Extracellular polysaccharides
GRAS: Generally regarded as safe
GS: Glucansucrase
4,6-α-GTs: 4,6-α-Glucanotransferases
HMM: High-molecular-mass
HPAEC-PAD: High-performance anion-exchange chromatography equipped with an ED40 pulsed amperometric detection (PAD) system
IMMO: Isomalto-/malto-oligosaccharide
IMMP: Isomalto-/malto-polysaccharide
LAB: Lactic acid bacteria
LMM: Low-molecular-mass
