# Supplementary material for: Structure–function relationships of family GH70 glucansucrase and 4,6-α-glucanotransferase enzymes, and their evolutionary relationships with family GH13 enzymes
Source: Cell Mol Life Sci. 2016 May 7;73(14):2681–706. doi: 10.1007/s00018-016-2245-7 (PMC4919382; doi:10.1007/s00018-016-2245-7)
Supplement: Supplementary file 1 — Supplementary material 1 (DOC 179 kb) [file 18_2016_2245_MOESM1_ESM.doc]

**Supplementary data**

**Multi-Author Review**

## Structure-function relationships of family GH70 glucansucrase and 4,6-α-glucanotransferase enzymes, and their evolutionary relationships with family GH13 enzymes

Xiangfeng Meng^1^, Joana Gangoiti^1^, Yuxiang Bai^1^, Tjaard Pijning^2^, Sander S. Van Leeuwen^1^ & Lubbert Dijkhuizen^1^*

*^1^ Microbial Physiology, Groningen Biomolecular Sciences and Biotechnology Institute (GBB), University of Groningen, Nijenborgh 7, 9747 AG Groningen, The Netherlands*

*^2^ Biophysical Chemistry, Groningen Biomolecular Sciences and Biotechnology Institute (GBB), University of Groningen, Nijenborgh 7, 9747 AG Groningen, The Netherlands*

__________

* Corresponding author: Lubbert Dijkhuizen; e-mail: [l.dijkhuizen@rug.nl](mailto:l.dijkhuizen@rug.nl)

**Table S1** Detailed information of family GH13 and GH70 enzymes used in the phylogenetic analysis.

| Enzymes | GeneBank accession | Strains | Polysaccharide |
| --- | --- | --- | --- |
| GH13  α-amylases | ACL70573.1 | *Halothermothrix orenii* H 168 | -- |
|  | CAQ88694.1 | *Escherichia fergusonii* ATCC 35469 | -- |
|  | WP_029392171.1 | *Escherichia coli* | -- |
|  | AEW62138.1 | *Klebsiella pneumoniae subsp. pneumoniae* HS11286 | -- |
|  | WP_045631742.1 | *Klebsiella pneumoniae* | -- |
|  | AAK89505.2 | *Agrobacterium fabrum* str. C58 | -- |
|  | EDV04833.1 | *Bacteroides intestinalis* DSM 17393 | -- |
|  | KKB50275.1 | *Parabacteroides goldsteinii* DSM 19448 | -- |
|  | EIK36872.1 | *Bacteroides fragilis* CL07T00C01 | -- |
|  | EOR99993.1 | *Bacteroides thetaiotaomicron* dnLKV9 | -- |
|  | WP_041556248.1 | *Odoribacter splanchnicus* | -- |
|  | KGB29922.1 | *Bacillus coagulans* | -- |
|  | CEO73502.1 | *Streptococcus pneumoniae* | -- |
|  | ETD97345.1 | *Streptococcus mitis* 21/39 | -- |
|  | AAB86961.1 | *Geobacillus stearothermophilus* | -- |
|  | AAA22235.2 | *Geobacillus stearothermophilus* | -- |
|  | AIV43245.1 | *Geobacillus stearothermophilus* | -- |
|  | WP_019418183.1 | *Anoxybacillus kamchatkensis* | -- |
|  | EFM12522.1 | *Paenibacillus curdlanolyticus* YK9 | -- |
|  | EWH20907.1 | *Bacillus licheniformis* S 16 | -- |
|  | WP_025807921.1 | *Bacillus licheniformis* | -- |
|  | WP_026828843.1 | *Exiguobacterium sibiricum* | -- |
|  | EEM21580.1 | *Bacillus thuringiensis serovar tochigiensis* BGSC 4Y1 | -- |
|  | EEK66557.1 | *Bacillus cereus* MM3 | -- |
|  | EEK72324.1 | *Bacillus cereus* AH621 | -- |
|  | EOO38153.1 | *Bacillus cereus* VDM019 | -- |
| GH70  GTFC-  like 4,6-  -α-GTs | WP_052702730.1 | *Paenibacillus beijingensis* | -- |
|  | AJE22990.1 | *Azotobacter chroococcum* NCIMB 8003 | -- |
|  | WP_029713886.1 | *Bacillus coagulans* | -- |
|  | AEH52441.1 | *Bacillus coagulans* 2-6 | -- |
|  | AJH79253.1 | *Bacillus coagulans* DSM 1 | -- |
|  | WP_017553304.1 | *Bacillus coagulans* | -- |
|  | WP_029141257.1 | *Bacillus coagulans* | -- |
|  | WP_047819242.1 | *Geobacillus sp.* 12AMOR1 | -- |
|  | WP_035322188.1 | *Bacillus kribbensis* | -- |
|  | WP_047390368.1 | *Exiguobacterium sp.* ZWU0009 | -- |
|  | WP_029342707.1 | *Exiguobacterium acetylicum* | -- |
|  | KNH34779.1 | *Exiguobacterium acetylicum* | -- |
|  | EZP59713.1 | *Exiguobacterium sp.* RIT341 | -- |
|  | ACB62096.1 | *Exiguobacterium sibiricum* 255-15 | -- |
|  | WP_028105602.1 | *Exiguobacterium undae* | -- |
|  | WP_026827371.1 | *Exiguobacterium sibiricum* | -- |
|  | AFS71545.1 | *Exiguobacterium antarcticum* B7 | -- |
|  | WP_026830256.1 | *Exiguobacterium antarcticum* | -- |
| GH70  GTFB-  like 4,6-  -α-GTs | AAU08014.2 | *Lactobacillus reuteri* 121 | -- |
|  | AAU08003.2 | *Lactobacillus reuteri* ML1 | -- |
|  | ABQ83597.1 | *Lactobacillus reuteri* DSM 20016 | -- |
|  | WP_050955745.1 | *Lactobacillus acidipiscis* | -- |
|  | WP_052661628.1_ | *Lactobacillus plantarum* | -- |
|  | EEI21226.1 | *Lactobacillus fermentum* ATCC 14931 | -- |
|  | EEX25696.1 | *Lactobacillus fermentum* 28-3-CHN | -- |
|  | KLD54475.1 | *Lactobacillus fermentum* | -- |
|  | EEI09591.1 | *Lactobacillus reuteri* MM2-3 | -- |
|  | WP_019251413.1 | *Lactobacillus reuteri* | -- |
|  | WP_052697219.1 | *Lactobacillus plantarum* | -- |
|  | AGO09550.1 | *Lactobacillus plantarum* 16 | -- |
|  | ABP88725.1 | *Lactobacillus reuteri* | -- |
|  | KOF04763.1 | *Lactobacillus reuteri* | -- |
|  | EGM52218.1 | *Lactobacillus salivarius* GJ-24 | -- |
|  | WP_053069107.1 | *Lactobacillus mucosae* | -- |
|  | WP_033607967.1 | *Lactobacillus plantarum* | -- |
|  | KNE73905.1 | *Lactobacillus delbrueckii* subsp. sunkii | -- |
|  | CCG90643.1 | *Pediococcus pentosaceus* IE-3 | -- |
|  | EOD02243.1 | *Lactobacillus delbrueckii* DSM 26046 | -- |
|  | WP_035162295.1 | *Lactobacillus delbrueckii* | -- |
|  | EFK31460.1 | *Lactobacillus delbrueckii* subsp. bulgaricus | -- |
|  | WP_035171046.1 | *Lactobacillus delbrueckii* | -- |
|  | WP_052933722.1 | *Lactobacillus delbrueckii* | -- |
|  | WP_025895575.1 | *Lactobacillus delbrueckii* | -- |
|  | KNZ37797.1 | *Lactobacillus delbrueckii* subsp. delbrueckii | -- |
|  | WP_035182758.1 | *Lactobacillus delbrueckii* | -- |
|  | ADQ61508.1 | *Lactobacillus delbrueckii* subsp. bulgaricus ND02 | -- |
| GH70 GSs | CCK33644.1 | *Lactobacillus animalis* TMW 1.971 | dextran |
|  | CCK33643.1 | *Lactobacillus curvatus* TMW 1.624 | dextran |
|  | AAU08008.1 | *Lactobacillus fermentum* KG3 | dextran |
|  | AAU08006.1 | *Lactobacillus parabuchneri* 33 | dextran |
|  | AAU08001.1 | *Lactobacillus reuteri* 180 | dextran |
|  | AAU08004.1 | *Lactobacillus reuteri* ML1 | mutan |
|  | AAU08015.1 | *Lactobacillus reuteri* 121 | reuteran |
|  | AAY86923.1 | *Lactobacillus reuteri* ATCC 55730 | reuteran |
|  | ABP88726.1 | *Lactobacillus reuteri* TMW 1.106 | dextran |
|  | AAU08011.1 | *Lactobacillus sakei* KG15 | dextran |
|  | BAF96719.1 | *Leuconostoc citreum* HJ-P4 | dextran |
|  | ACY92456.2 | *Leuconostoc citreum* B/110-1-2 | dextran |
|  | AIM52834.1 | *Leuconostoc citreum* ABK-1 | ND |
|  | ACA83218.1 | *Leuconostoc citreum* KM20 | ND |
|  | ACT20911.1 | *Leuconostoc lactis* EG001 | ND |
|  | AAB40875.1 | *Leuconostoc citreum* NRRL B-1299 | dextran |
|  | AAB95453.1 | *Leuconostoc citreum* NRRL B-1299 | dextran |
|  | AAD10952.1 | *Leuconostoc mesenteroides* NRRL B-512F | dextran |
|  | BAA90527.1 | *Leuconostoc mesenteroides* NRRL B-512F | dextran |
|  | CAB76565.1 | *Leuconostoc mesenteroides* NRRL B-1355 | dextran |
|  | CAB65910.2 | *Leuconostoc mesenteroides* NRRL B-1355 | alternan |
|  | AAG38021.1 | *Leuconostoc mesenteroides* B-742B | dextran |
|  | AAG61158.1 | *Leuconostoc mesenteroides* LCC4 | dextran |
|  | CAD22883.1 | *Leuconostoc citreum*NRRL B-1299 | dextran |
|  | CAD22883.1 | *Leuconostoc citreum* NRRL B-1299 | (α1→2) |
|  | CDX66896.1 | *Leuconostoc citreum* NRRL B-1299 | (α1→2) |
|  | CDX66895.1 | *Leuconostoc citreum* NRRL B-1299 | dextran |
|  | AAN38835.1 | *Leuconostoc mesenteroides* NRRL B-1501 | ND |
|  | AAS79426.1 | [*Leuconostoc mesenteroides* IBT-PQ](http://www.ncbi.nlm.nih.gov/Taxonomy/Browser/wwwtax.cgi?id=1245) | dextran |
|  | AAQ98615.2 | [*Leuconostoc mesenteroides* L0309](http://www.ncbi.nlm.nih.gov/Taxonomy/Browser/wwwtax.cgi?id=1245) | ND |
|  | ABC75033.1 | Leuconostoc mesenteroides 0326 | dextran |
|  | ABF85832.1 | *Leuconostoc mesenteroides* NRRL B-1299CB4 | dextran |
|  | AFP53921.1 | *Leuconostoc mesenteroides* KIBGE IB-22 | dextran |
|  | BAF62338.1 | *Streptococcus criceti* GTC242/HS-6 | mutan |
|  | AAA26898.1 | *Streptococcus downei* MFE 28 | dextran |
|  | AAC63063.1 | *Streptococcus downei* MFE 28 | mutan |
|  | AAC43483.1 | *Streptococcus gordonii* str.Challis substr.CH1 | dextran |
|  | BAA26114.1 | *Streptococcus mutans* | mutan |
|  | AAN58619.1 | *Streptococcus mutans* UA159 | dextran |
|  | AAN58705.1 | *Streptococcus mutans* UA159 | mutan |
|  | AAN58706.1 | *Streptococcus mutans* UA159 | mutan |
|  | BAA95201.1 | *Streptococcus oralis* ATCC10557 | dextran |
|  | BAF62337.1 | *Streptococcus orisuis* NUM 1001/JCM14035 | mutan |
|  | AAA26896.1 | *Streptococcus salivarius* ATCC 25975 | mutan |
|  | CAA77898.1 | *Streptococcus salivarius* ATCC 25975 | dextran |
|  | AAC41412.1 | *Streptococcus salivarius* ATCC 25975 | alternan |
|  | AAC41413.1 | *Streptococcus salivarius* ATCC 25975 | dextran |
|  | BAF43788.1 | *Streptococcus sanguinis* ATCC 10556 | dextran |
|  | BAA14241.1 | *Streptococcus sobrinus* | mutan |
|  | BAA02976.1 | *Streptococcus sobrinus* [ATCC 33478/OMZ176](http://www.ncbi.nlm.nih.gov/Taxonomy/Browser/wwwtax.cgi?id=1310) | mutan |
|  | BAC07265.1 | *Streptococcus sobrinus* B13N | Highly branched |
|  | AAX76986.1 | *Streptococcus sobrinus* B13N/OMZ176 | ND |
|  | ACK38203.1 | *Weissella cibaria* CMU | dextran |
|  | ADB43097.3 | *Weissella cibaria* LBAE-K39 | dextran |
|  | AKE50934.1 | *Weissella confusa* Cab3 | dextran |
|  | CCF30682.1 | *Weissella confusa* LBAE C39-2 | dextran |
|  | AHU88292.1 | [*Weissella confusa* VTT E-90392](http://www.ncbi.nlm.nih.gov/Taxonomy/Browser/wwwtax.cgi?id=1583) | dextran |

ND, not determined.
